# An Adapted Cardioprotective Diet with or Without Phytosterol and/or Krill Oil Supplementation in Familial Hypercholesterolemia: Results of a Pilot Randomized Clinical Trial

**DOI:** 10.3390/nu17122008

**Published:** 2025-06-15

**Authors:** Erlon Oliveira de Abreu-Silva, Rachel Helena Vieira Machado, Bianca Rodrigues dos Santos, Flávia Cristina Soares Kojima, Renato Hideo Nakagawa Santos, Karina do Lago Negrelli, Letícia Barbante Rodrigues, Pedro Gabriel Melo de Barros e Silva, Andressa Gusmão de Lima, João Gabriel Sanchez, Fernanda Jafet El Khouri, Ângela Cristine Bersch-Ferreira, Adriana Bastos Carvalho, Thaís Martins de Oliveira, Maria Cristina Izar, Geni Rodrigues Sampaio, Nágila Raquel Teixeira Damasceno, Marcelo Macedo Rogero, Elizabeth Aparecida Ferraz da Silva Torres, Flávia De Conti Cartolano, Julia Pinheiro Krey, Patrícia Vieira de Luca, Cristiane Kovacs Amaral, Elisa Maia dos Santos, Rodrigo Morel Vieira de Melo, Eduardo Gomes Lima, André de Luca dos Santos, Thiago Gomes Heck, Ana Paula Perillo Ferreira Carvalho, Silvia Bueno Garofallo, Alexandre Biasi Cavalcanti, Aline Marcadenti

**Affiliations:** 1 Hcor Research Institute, São Paulo 04004-030, SP, Brazil; erlon@terra.com.br (E.O.d.A.-S.); rhelena@hcor.com.br (R.H.V.M.); biasantos@hcor.com.br (B.R.d.S.); fkojima@hcor.com.br (F.C.S.K.); rnakagawa@hcor.com.br (R.H.N.S.); knegrelli@hcor.com.br (K.d.L.N.); lbarbante@hcor.com.br (L.B.R.); drpedrobarros80@gmail.com (P.G.M.d.B.e.S.); aglima@hcor.com.br (A.G.d.L.); jgabriel@hcor.com.br (J.G.S.); fjkhouri@hcor.com.br (F.J.E.K.); abiasi@hcor.com.br (A.B.C.); 2 Division of Health Care Sciences, Dresden International University, 01307 Dresden, Germany; 3 Department of Education and Research, BP—A Beneficência Portuguesa de São Paulo, São Paulo 01323-001, SP, Brazil; angelacbferreira@gmail.com; 4 Carlos Chagas Filho Biophysics Institute, Federal University of Rio de Janeiro, Rio de Janeiro 21941-599, RJ, Brazil; carvalhoab@biof.ufrj.br; 5 National Institute of Cardiology, Rio de Janeiro 22240-006, RJ, Brazil; 6 Graduate Program in Genetics and Molecular Biology, Federal University of Rio Grande do Sul, Porto Alegre 91501-970, RS, Brazil; thaismartins023@gmail.com; 7 Paulista School of Medicine, Federal University of São Paulo, São Paulo 04024-002, SP, Brazil; cristina.izar@unifesp.br; 8 Department of Nutrition, School of Public Health, University of São Paulo, São Paulo 01246-904, SP, Brazil; genirs@usp.br (G.R.S.); nagila@usp.br (N.R.T.D.); mmrogero@usp.br (M.M.R.); eatorres@usp.br (E.A.F.d.S.T.); fdeconti@gmail.com (F.D.C.C.); 9 Hcor Nutrition Service, São Paulo 04004-030, SP, Brazil; jkrey@hcor.com.br; 10 Brazilian Association of Familial Hypercholesterolemia, São Paulo 04044-903, SP, Brazil; pvl@ahfcolesterol.org; 11 Ambulatório de Nutrição Clínica, Instituto Dante Pazzanese de Cardiologia, São Paulo 04012-180, SP, Brazil; cristiane.kovacs@dantepazzanese.org.br; 12 Graduate Program in Cardiovascular Sciences, National Institute of Cardiology, Rio de Janeiro 22240-006, RJ, Brazil; elisamaia80@gmail.com; 13 Graduate Program in Medicine and Health, Federal University of Bahia, Salvador 40026-010, BA, Brazil; rodrigo.morel@ufba.br; 14 Hospital Ana Nery, Salvador 40320-010, BA, Brazil; 15 Atherosclerosis Clinical Unit, Hospital das Clínicas Heart Institute, School of Medicine, University of São Paulo, São Paulo 05403-000, SP, Brazil; eduglima@yahoo.com.br; 16 Dasa Cardiology Unit (Hospital 9 de Julho), São Paulo 01409-002, SP, Brazil; 17 Clinical Research Center, Hospital São José, Criciúma 88811-500, SC, Brazil; dedelucabr@hotmail.com; 18 Graduate Programs in Integral Health Care and in Mathematical and Computational Modeling, Regional University of the Northwest of the State of Rio Grande do Sul (UNIJUÍ), Ijuí 98700-000, RS, Brazil; thiago.heck@unijui.edu.br; 19 Hypertension Unit and Health and Nutrition Research Improvement Group, Clinical Hospital, Federal University of Goiás, Goiânia 74605-020, GO, Brazil; anapaulaperillo@ufg.br; 20 Graduate Program in Health Sciences (Cardiology), Rio Grande do Sul Cardiology Institute, University Foundation of Cardiology, Porto Alegre 90040-371, RS, Brazil; silviagarofallo@feevale.br; 21 School of Medicine, Feevale University, Novo Hamburgo 93510-235, RS, Brazil; 22 Graduate Program in Public Health, University of São Paulo, São Paulo 01246-904, SP, Brazil

**Keywords:** hypercholesterolemia, diet, healthy, fatty acids, phytosterols, omega-3, pilot projects

## Abstract

**Background/Objectives:** Familial hypercholesterolemia (FH) is an increasingly common inherited disorder that increases cardiovascular risk. Despite the importance of lifestyle interventions, adherence to a healthy diet among individuals with FH remains suboptimal. This pilot, multicenter, double-blind, placebo-controlled randomized trial aimed to evaluate the feasibility and preliminary effects of a culturally adapted cardioprotective diet (DICA-FH), alone or in combination with phytosterol and/or krill oil supplementation, on lipid parameters in Brazilian adults with probable or definitive FH. **Methods:** Between May and August 2023, 58 participants were enrolled across nine Brazilian centers and randomized (1:1:1:1) into four groups: DICA-FH + phytosterol placebo + krill oil placebo; DICA-FH + phytosterol 2 g/day + krill oil placebo; DICA-FH + phytosterol placebo + krill oil 2 g/day; and DICA-FH + phytosterol 2 g/day + krill oil 2 g/day. Interventions lasted 120 days. The primary outcomes were mean low-density lipoprotein cholesterol (LDL-c) and lipoprotein(a) (Lp[a]) levels, as well as adherence to treatment at follow-up. Secondary outcomes included mean levels of other lipids, frequency of adverse events, and assessment of protocol implementation components. All data were presented separately for the allocation groups: phytosterol vs. placebo and krill oil vs. placebo. **Results:** Mean age was 54.5 ± 13.7 years, and 58.6% were women. Both adherence to protocol (91.8% attendance; 79.1% investigational product intake) and retention (86.2%) were high. No significant differences between groups were found for LDL-c or Lp(a). However, regardless of allocation to active supplementation or placebo, a significant reduction in Lp(a) concentrations was observed following the DICA-FH intervention (median difference: −3.8 mg/dL [interquartile range: −7.5 to −1.2]; *p* < 0.01). Significant reductions in oxidized LDL (LDL-ox) and LDL-ox/LDL-c ratio were also observed in the overall sample (*p* < 0.01). Although not statistically significant, all groups showed improvements in diet quality after 120 days. No serious adverse events related to the interventions were reported. Additionally, most protocol implementation components were successfully achieved. **Conclusions:** The DICA-FH strategy, with or without supplementation, was safe and well-tolerated. Although not powered to detect clinical efficacy (which is acceptable in exploratory pilot trials), the study supports the feasibility of a larger trial and highlights the potential of dietary interventions in the management of HF.

## 1. Introduction

Familial hypercholesterolemia (FH) is a highly prevalent inherited cardiovascular disorder, affecting approximately one in three hundred individuals in the general population and about one in thirty among those with ischemic heart disease [1]. The heterozygous form of FH is transmitted in an autosomal dominant manner, primarily due to pathogenic variants in the *LDLR* (LDL receptor), *APOB* (Apolipoprotein B), and *PCSK9* (Proprotein convertase subtilisin/kexin type 9) genes. Less frequently, variants in *APOE* (Apolipoprotein E) and *STAP1* (signal transducing adaptor family member 1) genes have also been implicated [2]. Despite being relatively common, reports of underdiagnosis are frequent even in developed countries, as are high rates of individuals receiving inadequate treatment [3].

Achieving optimal levels of low-density lipoprotein cholesterol (LDL-c) and lipoprotein(a) [Lp(a)] is a central goal in the management of FH, often requiring a combination of pharmacological therapies [4,5]. Nonetheless, lifestyle modifications are considered a fundamental part of treatment and should accompany pharmacological therapy [4]. Within this context, dietary interventions play a critical role in supporting medication-based approaches. However, evidence suggests that individuals with FH often perceive dietary changes as less important than pharmacological treatment [6].

While meta-analyses have identified a range of potential dietary strategies for managing FH [7,8], current clinical guidelines primarily recommend the Mediterranean diet [9,10]. Despite its established benefits, adherence to this dietary pattern tends to be low among individuals with FH who live outside the Mediterranean region [11]. The Brazilian Cardioprotective Diet (DICA Br—*DIeta CArdioprotetora Brasileira*), which promotes an accessible and sustainable eating pattern based on national dietary guidelines [12], may enhance adherence to healthy eating habits by aligning with local food culture. However, it has not demonstrated significant effects on LDL-c levels in secondary cardiovascular prevention settings [13].

Phytosterol supplementation, when combined with a healthy dietary pattern, is recommended as an adjunctive therapy for individuals with FH [9,14]. In contrast, omega-3 polyunsaturated fatty acids (n-3 PUFAs) derived from fish oil should be used with caution [9,14] as they may raise LDL-c levels [15], despite their potential to improve Lp(a) concentrations [16]. Krill oil, which contains lower levels of n-3 PUFAs, has been proposed as a safer alternative to fish oil due to its enhanced bioavailability [17]; however, its efficacy has not yet been assessed in individuals with FH.

Adapting the DICA Br to the context of FH—by emphasizing affordable foods and nutrients with known lipid-lowering effects—and assessing its effectiveness with or without the use of dietary supplements, could not only broaden the spectrum of nutritional strategies available for this population but also support the development of approaches to improve dietary adherence among individuals with FH. Therefore, the primary objective of this pilot study was to evaluate the preliminary results of an adapted DICA Br diet, alone or in combination with phytosterol and/or krill oil supplementation, in individuals with a probable or definitive diagnosis of FH, in order to assess the feasibility of conducting a larger randomized clinical trial in the future.

## 2. Materials and Methods

### 2.1. Study Design and Ethical Considerations

The study protocol was previously published and described in detail [18]. DICA-FH is a national, multicenter, double-dummy placebo-controlled randomized pilot trial with a superiority factorial design, conducted in parallel groups, with participants allocated equally across four arms (1:1:1:1). The pilot trial was conducted at nine sites across Brazil between May and December 2023, under the coordination of the Hcor Research Institute (IP-Hcor, São Paulo, Brazil). The study is registered on ClinicalTrials.gov (Identifier: NCT05695937) and has been assigned a World Health Organization Universal Trial Number (WHO-UTN: U1111-1296-7102). Ethical approval was granted by the Institutional Review Board at Hcor, as well as by the ethics committees of all participating centers. The study followed ethical principles for research involving human subjects, and written informed consent was obtained from all participants prior to enrollment, with the process conducted by trained research staff. Furthermore, the study followed the Consolidated Standards of Reporting Trials (CONSORT) guidelines for pilot and feasibility trials (Table S1, Supplementary Material) [19].

### 2.2. Participants

Researchers included men and women aged 20 years or older who had a definitive or probable diagnosis of FH, as defined by the Dutch Lipid Clinic Network (Dutch MEDPED) criteria [20]. To be eligible, participants had to be undergoing moderate- to high-intensity lipid-lowering therapy for at least six weeks, with treatment regimens specified in detail in the study protocol [18]. Researchers excluded individuals with a ‘possible’ FH diagnosis according to Dutch MEDPED criteria, serum triglyceride levels ≥500 mg/dL, or hypercholesterolemia secondary to other conditions. Additional exclusion criteria were as follows: food allergies; contraindications to phytosterol use; HIV infection under treatment or AIDS; chronic inflammatory diseases; liver disease or chronic kidney disease requiring dialysis; active cancer or a life expectancy of less than six months; acute coronary syndrome within the past 60 days; substance use disorder (including alcohol); chronic use of anti-inflammatory, anticonvulsant, or immunosuppressive medications; use of PCSK9 inhibitors; pregnancy or breastfeeding; inability to perform anthropometric assessments due to wheelchair use; body mass index (BMI) ≥ 40 kg/m^2^; current use of dietary supplements that could affect study outcomes; participation in other randomized clinical trials; and refusal to provide informed consent [18].

Study staff at each center identified eligible participants primarily through cardiology and specialized outpatient clinics and invited them to participate in the trial. They also included volunteers who responded to invitations shared on social media.

### 2.3. Randomization and Blinding

The IP-Hcor team developed a centralized randomization system specifically for this study (https://servicos3.hcor.com.br/redcap/ accessed on 12 December 2024). Researchers performed a stratified randomization (by center sites) in blocks of varying sizes, maintaining a 1:1:1:1 allocation ratio. A validated software generated the randomization list, and only the study coordinator had access to the sequence to ensure allocation concealment. Furthermore, the randomization strategy was designed using unique coding to maintain blinding across all allocation groups, in case unblinding became necessary due to a serious adverse event potentially related to the intervention. Investigators enrolled participants by completing a standardized clinical form in the electronic case report form (eCRF). Once all eligibility criteria were confirmed and the informed consent form was signed by the participant, the system generated the randomization assignment [18]. The investigators, study participants, caregivers, and outcome evaluators were blinded to the investigational products [18].

### 2.4. Study Interventions

Study participants were assigned to one of four treatment groups: (1) Control: DICA-FH + phytosterol placebo + krill oil placebo (*n* = 16); (2) DICA-FH + phytosterol 2 g/day + krill oil placebo (*n* = 13); (3) DICA-FH + phytosterol placebo + krill oil 2 g/day (*n* = 15); or (4) DICA-FH + phytosterol 2 g/day + krill oil 2 g/day (*n* = 14). The study protocol did not incorporate any co-interventions, such as increased physical activity, stress reduction measures, or other behavioral modifications [18].

The study protocol provides a detailed description of the process used to adapt the DICA Br to the context of FH [18]. Briefly, DICA Br is a nutritional strategy tailored for the Brazilian population, endorsed by the Brazilian Ministry of Health as a method for managing cardiometabolic risk factors [12,13,21]. This dietary approach categorizes foods into three groups based on nutritional principles such as nutrient and energy density, as well as the NOVA classification system [22]. The food groups were color-coded to align with the Brazilian flag, aiding comprehension and adherence: the green group (fresh and minimally processed foods rich in cardioprotective nutrients, such as fruits, vegetables, beans, and low-fat dairy) should be consumed in larger quantities because this is the color that occupies the largest area on the Brazilian flag. The yellow group (higher-energy foods such as cereals, nuts, vegetable oils, and honey) should be consumed less frequently; and the blue group (foods high in animal protein and fat, including meats, cheeses, and eggs) should be eaten in smaller portions because this is the color that occupies the smallest area on the Brazilian flag. Ultra-processed foods were strongly discouraged and placed in the red group (not represented by a color on the Brazilian flag). The DICA Br approach can be implemented either qualitatively or prescriptively, with this study adopting the qualitative approach to facilitate guidance by multidisciplinary teams [12,18].

The researchers adapted and incorporated the principles of the Portfolio Diet, a plant-based dietary pattern developed to reduce serum cholesterol levels, into DICA Br [23,24]. This diet focuses on consuming foods (or nutrients) with cholesterol-lowering properties, such as oilseeds, vegetable proteins (soybeans and legumes), soluble fibers (oats, barley, psyllium, eggplant, okra, apple, orange, and berries), plant sterols (fruits and vegetables), and monounsaturated fatty acids (olive oil, sunflower oil, canola oil, avocado) [25]. The researchers classified the foods emphasized in the Portfolio Diet according to DICA Br colors (oilseeds and monounsaturated fatty acids in the yellow group; vegetable proteins, soluble fibers, and plant sterols in the green group), and they encouraged participants to consume them based on availability and whenever possible.

All participants received guidance on the DICA Br adapted for the FH context (DICA-FH) and were provided with educational materials designed to enhance their understanding and adherence to the diet [18].

Group 1 (Control): The participants received guidance on DICA-HF along with phytosterol and krill oil placebos, which replicated the color, smell, taste, and appearance of the active ingredients, containing the same number of capsules as the active treatments.

Group 2: The researchers provided Group 2 with guidance on DICA-HF along with 2 g/day of phytosterols (two capsules of 1 g) and a krill oil placebo (four capsules of 500 mg).

Group 3: Along with the DICA-FH guidance, the researchers gave Group 3 a phytosterol placebo (two capsules of 1 g) and 2 g/day of krill oil (four capsules of 500 mg).

Group 4: In addition to the DICA-FH guidance, the researchers provided Group 4 with 2 g/day of phytosterols (two capsules of 1 g) and 2 g/day of krill oil (four capsules of 500 mg).

The researchers provided all groups with the same instructions regarding the storage and dosage of the investigational products, advising participants to take them preferably within separate main meals (phytosterols/placebos at lunch and krill oil/placebos at dinner). Additionally, the researchers instructed participants to refrain from using any other food supplements during the study.

The nutritional composition of the investigational products (phytosterol Vegapure^®^ 95 FF; krill oil Superba™ Boost; and soybean oil placebo) was previously described on the study protocol [18].

### 2.5. Outcomes

The study protocol describes in detail all the methods used to evaluate primary and secondary outcomes [18].

- Primary outcomes after the 120-day follow-up. (1) For phytosterol vs. placebo groups: mean LDL-c level. For krill oil vs. placebo groups: mean Lp[a] level. (2) Adherence to treatment assessed by presence in appointments, diet quality according to the Cardiovascular Health Diet Index (CHDI) [26], proportion of consumed investigational products, and analysis of plasma phytosterol and erythrocyte fatty acid levels [18].
- Secondary outcomes after the 120-day follow-up. (1) Means of total cholesterol, high density lipoprotein cholesterol (HDL-c), serum triglycerides, non-HDL cholesterol, very-low density lipoprotein cholesterol (VLDL-c), total cholesterol/HDL-c ratio, LDL-c/HDL-c ratio, atherogenic index, triglycerides/HDL-c ratio, oxidized LDL (ox-LDL), and apolipoproteins (Apo[s]) A-I and B. (2) Frequency of adverse events throughout the study. (3) Components of protocol implementation evaluated based on loss-to-follow-up rates, recruitment rates within the expected time frame, and the selection and adjustments of remote platforms/media for site training and study participants.

### 2.6. Study Procedures

The study coordinators conducted remote training for all investigators, following the standardized procedures outlined in the study manual. The researchers described previously in the study protocol and in detail all sociodemographic, clinical, lifestyle, and anthropometric variables, along with all procedures conducted during each study visit [18]. The research team invited participants to join the study either in person or by telephone. They instructed participants to fast for 12 h, avoid alcohol for 72 h before the initial visit, and refrain from engaging in vigorous physical activity. In addition to collecting a blood sample, the researchers also collected a saliva sample for whole exome sequencing, in accordance with the study protocol [18]. Follow-up visits took place at 40, 80, and 120 days (final visit, during which new blood samples were collected). Between appointments, participants completed online forms at home with questions about supplement use and the occurrence of adverse events. They also received an individualized link via email or SMS to complete the 24-h dietary recall (R24h). During all visits, the researchers instructed participants to keep the packaging until the end of the study.

### 2.7. Sample Size

All procedures related to the sample size calculation required for conducting a large-scale randomized clinical trial (*n* = 268) are described in detail in the study protocol [18]. For the pilot study, the researchers aimed to include between 48 and 76 participants (approximately 30–40% of the full study sample), based on an estimated overall adherence rate of 80% to the protocol, with a precision of ±9.5% and a 90% confidence interval (CI).

### 2.8. Statistical Analysis

The analyses followed the modified intention-to-treat principle, considering only complete cases. Missing values for the target visit were not imputed. Continuous variables were summarized as mean and standard deviation (for normally distributed data) or as median and interquartile range (for non-normal data). Categorical variables were expressed as absolute and relative frequencies. All data were presented separately for the allocation groups: phytosterol vs. placebo and krill oil vs. placebo.

For continuous outcomes with a normal distribution, unpaired Student’s *t*-tests were used to compare groups, with results reported as mean differences and 95% CI. Within-group comparisons were performed using paired Student’s *t*-test. For non-normal distribution, the Wilcoxon–Mann–Whitney test was applied to compare groups, with the Hodges–Lehmann estimator and 95% CI reported. Within-group changes were assessed using the Wilcoxon signed-rank test.

To assess adherence, we estimated the percentage of attendance at visits (remote and/or in-person) and the 90% CI, considering the entire sample without dividing the allocated groups. The same approach was applied to adherence to supplement intake. We evaluated adverse events by comparing groups using the chi-square test.

The significance level was set at 5%, without adjustment for multiple comparisons. Analyses were performed using R software, version 4.3.2 (R Foundation for Statistical Computing).

## 3. Results

### 3.1. Recruitment and Participant Characteristics

**Figure 1:**
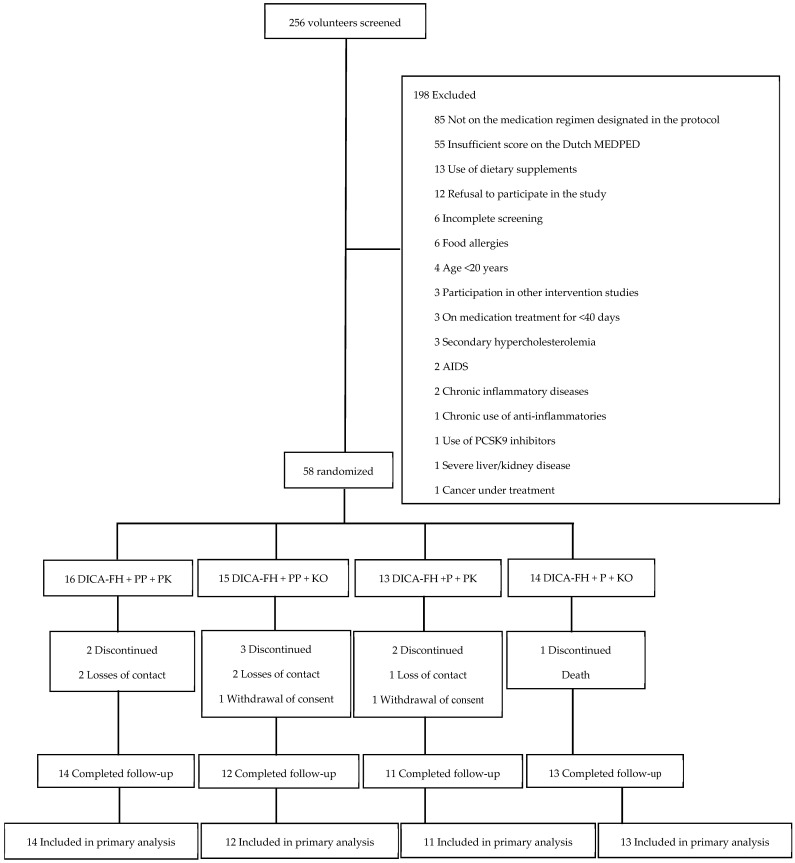
Flowchart of the study. AIDS: Acquired Immunodeficiency Syndrome; PCSK9: proprotein convertase subtilisin/kexin type 9; DICA-FH: *DIeta CArdioprotetora Brasileira* adapted to familial hypercholesterolemia; PP: phytosterol placebo; PK: krill oil placebo; P: active phytosterol; KO: active krill oil.

Between May and August 2023, a total of 256 individuals were screened. Of these, 198 were excluded for not meeting the eligibility criteria—most commonly due to not being on the medication regimen specified in the study protocol or not reaching the minimum required score on the Dutch MEDPED criteria (Figure 1). A total of 58 men and women with a probable or definitive diagnosis of FH, based on the Dutch MEDPED criteria, were enrolled across nine participating sites in four regions of Brazil (South, Southeast, Central–West, and Northeast). Among them, 16 participants were assigned to the control group (phytosterol placebo + krill oil placebo), 15 to the phytosterol placebo + active krill oil krill, 13 to the active phytosterol + krill oil placebo, and 14 to the combined group (active phytosterol + krill oil).

The sample had a higher proportion of women (58.6%) and predominantly white participants. Additionally, 36.2% were from lower socioeconomic strata, with a reported monthly household income between USD 189.18 and USD 698.31. The mean age was 54.5 ± 13.7 years, and 13.8% of participants were unaware of any family history of FH. The mean LDL-c was 132.7 ± 52.5 mg/dL, and the median Lp(a) level was 31.4 mg/dL (interquartile range [IQR] 17.2–62.1). A total of 57.1% had a history of previous cardiovascular events, including 20 individuals who had experienced a myocardial infarction prior to enrollment.

Table 1a,b present the baseline characteristics of participants according to the phytosterol (active vs. placebo) and krill oil (active vs. placebo) groups. Overall, the groups were balanced in terms of baseline characteristics and concurrent lipid-lowering therapy (Tables S2 and S3, Supplementary Material). At baseline, 48 participants (82.8%) were receiving high-potency lipid-lowering treatment. Detailed baseline and treatment data by study arm are available in the Supplementary Material (Tables S4 and S5).

Among the 57 participants with an available Dutch MEDPED score (one participant qualified via a medical report), the mean score was 8.9 ± 3.6. Of these, 63.2% were classified as having a probable diagnosis and 36.8% as having a definitive diagnosis of FH. Whole-exome sequencing identified pathogenic variants in the *APOB* gene in five individuals, in *ABCG8* (ATP binding cassette subfamily G member 8) gene in one individual, and in *LDLR* in twenty-two individuals. Variants of uncertain significance were found in five individuals—four in the *LDLR* gene and one in *PCSK9*. No FH-related genetic mutations were identified in twenty-five participants. Table S6 of the Supplementary Material presents the genetic variants identified in the evaluated sample.

Among the thirty-six participants with Dutch MEDPED scores of 6–8 (indicative of probable FH), fifteen had pathogenic FH-related mutations, three had variants of uncertain significance, and eighteen had no detectable mutations. Of the twenty-one participants with scores > 8 (definitive FH), twelve had confirmed pathogenic mutations, two had uncertain variants, and seven had no identifiable mutations. The participant enrolled via medical documentation (without a Dutch MEDPED score) was found to have a variant in the *LDLR* gene.

### 3.2. Retention

All participants received the intervention according to their assigned groups. In the control group, two participants were lost to follow-up (reasons: loss of contact); in the phytosterol placebo + active krill oil group, three participants did not complete the study (two due to loss of contact and one due to withdrawal of consent); in the active phytosterol + krill oil placebo group, two participants were lost to follow-up due to loss of contact and withdrawal of consent; and in the combined group, one participant did not complete the study due to death.

Retention rates in each group were, respectively: 87.5%, 80%, 84.6%, and 92.8%. The study flowchart is presented in Figure 1.

### 3.3. Primary Outcomes

#### 3.3.1. LDL-c and Lp(a)

Table 2 presents the within- and between-group comparisons of LDL-c and Lp(a) concentrations. After 120 days of intervention, no statistically significant differences in LDL-c were observed between the phytosterol subgroups (placebo vs. active) or between the krill oil subgroups (placebo vs. active). Similarly, Lp(a) concentrations did not differ significantly between the active and placebo subgroups for either phytosterol or krill oil. However, within-group analyses revealed significant median reductions in Lp(a) levels in the phytosterol placebo subgroup, with a 10.9% decrease (*p* = 0.03), and in the krill oil placebo subgroup, which showed a 16.1% reduction (*p* < 0.001).

When analyzing all participants collectively, regardless of allocation to active supplementation or placebo, a significant reduction in Lp(a) concentrations was observed following the DICA-FH intervention. Among the 48 individuals included in this analysis, median Lp(a) levels decreased from 31.4 mg/dL (17.2–62.1) at baseline to 26.9 mg/dL (15.6–53.2) after follow-up (median difference: −3.8 mg/dL [interquartile range −7.5 to −1.2]; *p* < 0.01).

#### 3.3.2. Adherence

- Attendance at visits and investigational product consumption:

At the end of the study, the mean attendance rate across all scheduled visits was 91.8% (90%CI 87.1–96.6), with no significant differences observed between the study groups (control group: 95.3% ± 13.6; phytosterol placebo + active krill oil group: 86.7% ± 28.1; active phytosterol + krill oil placebo group: 90.4% ± 24.0; active phytosterol + krill oil group: 94.6% ± 20.0; *p* = 0.78).

**Figure 2:**
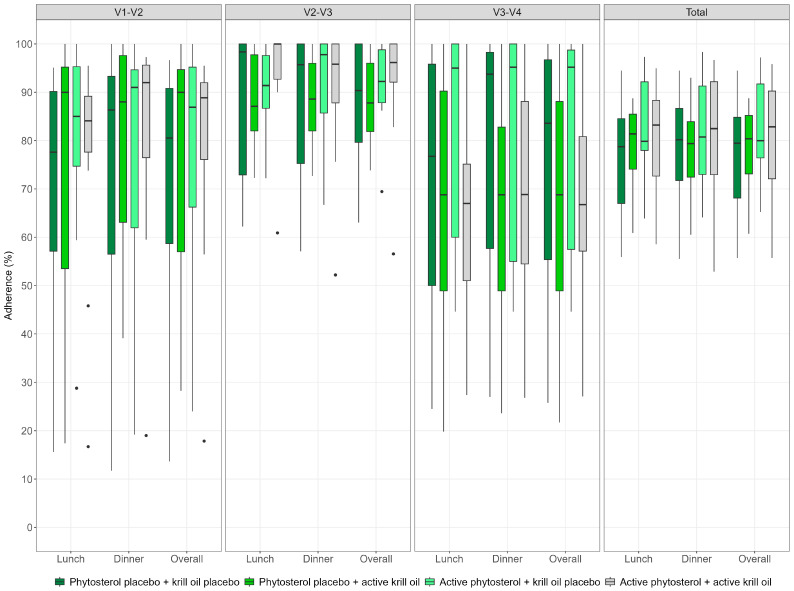
Proportion of investigational products consumed by study group and by study period/visit. V1–V2: period between visit one and visit two; V2–V3: period between visit two and visit three; V3–V4: period between visit three and visit four (final).

Figure 2 presents the proportion of investigational products consumed by study group and by study period/visit. There were no significant differences in consumption between the groups (*p* = 0.75). Considering the full sample, the overall mean intake of investigational products was 79.1% (90%CI 76.3–82.0), with no meaningful difference between consumption at lunch (78.8% [90%CI 76–81.6]) and dinner (79.5% [90%CI 76.5–82.5]).

- Diet quality:

After 120 days, no significant differences were observed in the CHDI total score or its individual components within the krill oil group. In the phytosterol group, a significant difference was found between the placebo and active phytosterol subgroups regarding fish consumption after follow-up (difference: active phytosterol—placebo = 2.34 points [0.06; 4.62]; *p* = 0.04). However, there were no significant differences in the CHDI total score or in the remaining components between the subgroups (Table 3). Although not statistically significant, all groups showed improvements in overall diet quality after 120 days.

- Plasma phytosterol analysis:

Table 4 presents plasma phytosterol concentrations before and after the interventions across groups, as well as values adjusted for total cholesterol and LDL-c levels. In the phytosterol group, the concentrations of stigmasterol, stigmasterol/total cholesterol, and stigmasterol/LDL-c were significantly higher in the active phytosterol subgroup compared to the placebo subgroup (respective median differences: 0.28 µg/mL [0.10; 0.48], *p* < 0.01; 0.12 µg/mg [0.05; 0.22], *p* < 0.01; and 0.20 µg/mg [0.08; 0.39], *p* < 0.01, despite baseline medians differing between subgroups). No significant differences were observed between subgroups for campesterol or beta-sitosterol concentrations.

In the krill oil group, there were no significant differences between subgroups in plasma phytosterol concentrations or in their cholesterol-adjusted values. Regardless of supplementation or placebo use, the DICA-FH intervention appeared to modulate the concentration of several plasma phytosterol biomarkers in the subsample for which these parameters were assessed (*n* = 40). A detailed description of these changes is provided in the Supplementary Material (Table S7).

- Erythrocyte fatty acid analysis:

Table 5 and Table S8 (Supplementary Material) show the concentrations of various erythrocyte fatty acids before and after the interventions according to the study groups. In the phytosterol group, a significant difference was observed in the concentration of cis-8,11,18 eicosatrienoic acid (C20:3 n6) at the end of follow-up between subgroups (median difference: active phytosterol—placebo = 0.25% [0.02; 0.48]; *p* = 0.03). In the krill oil group, significant differences were found in docosahexaenoic acid (DHA, C22:6 n3) concentrations (median difference: active krill oil—placebo = 0.79% [0.02; 1.56]; *p* = 0.01) and in the omega-3/omega-6 ratio (median difference: active krill oil—placebo = 0.05 [0.01; 0.09]; *p* = 0.01). No other significant between-group differences were observed for the remaining fatty acids in either the phytosterol or krill oil groups.

Independent of whether participants received supplementation or placebo, the DICA-FH intervention influenced the levels of various erythrocyte fatty acids in the subgroup of individuals evaluated for these parameters (*n* = 47). Further details on these changes are provided in the Supplementary Material (Table S9).

### 3.4. Secondary Outcomes

#### 3.4.1. Clinical Lipid Profile and Anthropometric Markers

After 120 days of intervention, no significant differences were observed in clinical lipid profile markers between subgroups within either the phytosterol or krill oil groups (Table S10, Supplementary Material). Regardless of treatment group, the DICA-FH intervention significantly reduced HDL-c concentrations in the total sample assessed (*n* = 50), with levels decreasing from 51.5 mg/dL ± 12.03 at baseline to 47.99 mg/dL ± 10.99 at follow-up (mean difference: −3.51 mg/dL [−5.88; −1.15]; *p* < 0.01).

Within the phytosterol group, no significant differences were found between subgroups regarding body weight, BMI, or waist circumference after follow-up (n). In contrast, within the krill oil group, the mean BMI was significantly lower in the placebo subgroup compared to the active krill oil subgroup at 120 days (median difference: krill oil—placebo = 2.91 kg/m^2^ [0.60; 5.23]; *p* = 0.01), although baseline means differed between the subgroups. No significant differences were found in body weight or waist circumference within the krill oil group.

#### 3.4.2. LDL-ox and Apolipoproteins

Table S11 (Supplementary Material) presents the values of LDL-ox, the LDL-ox/LDL-c ratio, and the concentrations of Apo(s) B and A-I at baseline and at the end of the study. No significant differences were found in these biomarkers between placebo and active supplementation subgroups. However, in the total sample (*n* = 50), the DICA-FH intervention significantly reduced LDL-ox concentrations, from 0.197 (0.144–0.252) at baseline to 0.155 (0.11–0.211) at 120-day follow-up (mean difference: −0.034 [−0.059; −0.014]; *p* < 0.01). A significant reduction was also observed in the LDL-ox/LDL-c ratio, which decreased from 0.162 (0.126–0.199) to 0.132 (0.099–0.163) (mean difference: −0.028 [−0.047; −0.010]; *p* < 0.01), regardless of supplementation status.

#### 3.4.3. Adverse Events

No significant differences were observed in the rate of adverse events reported during the study across the four randomized groups (Table S12, Supplementary Material). One serious adverse event (death) was reported and adjudicated during the study; however, it was deemed unrelated to the study interventions. At the end of the intervention period, no significant differences were found in aspartate aminotransferase and alanine aminotransferase levels between the phytosterol (active vs. placebo) or between the krill oil (active vs. placebo) subgroups (Table S13, Supplementary Material). These findings were consistent when the four randomization groups were analyzed independently. 

#### 3.4.4. Protocol Implementation Components

The overall loss to follow-up rate in the study was 13.8%, which was slightly higher than the 10% rate anticipated in the sample size calculation for the larger clinical trial, as detailed in the study protocol [18], with no significant differences among study groups. The expected recruitment range of 48 to 76 participants within four months was achieved (*n* = 58). Regarding the weekly food intake monitoring form, participant adherence to completion was low (10%). Similarly, adherence to the questionnaires used to monitor investigational product intake and adverse events—sent via SMS links—was also low, at 45%. Despite these limitations, the decentralized monitoring strategy enabled the identification of 83% of the adverse events reported during the study, which were subsequently reviewed and supplemented during site visits with the investigators. The platforms used for training and data collection across participating centers received a very positive overall evaluation.

## 4. Discussion

This pilot study, which evaluated DICA-FH with or without supplementation of phytosterols and/or krill oil in Brazilian individuals with a probable or definitive diagnosis of FH, demonstrated preliminary results regarding the impact of the interventions on various biomarkers, particularly those of clinical relevance in the context of the disease (LDL-c and Lp[a]). Moreover, the study suggests that the interventions are safe, as no differences were observed in the reporting or biochemical parameters of adverse events between groups. Considering the overall assessment of adherence indicators and the proposed protocol implementation, this pilot study also demonstrated the feasibility of conducting a larger-scale clinical trial at a national level.

The evaluated sample partially reflected the demographic characteristics identified in a larger study conducted in Brazil, which reported a higher age-weighted FH prevalence in women (supporting our findings) and in mixed-race and Black populations [27]. Given that a greater proportion of White individuals were enrolled in our study, this highlights the need to improve recruitment strategies in a broader clinical trial to ensure greater representativeness and population diversity. The higher frequency of pathogenic mutations identified in the *LDLR* gene is also consistent with the literature [28,29]. Although more than 80% of the sample was undergoing high-intensity lipid-lowering therapy, the mean LDL-c levels remained above the therapeutic targets recommended in clinical guidelines, regardless of participants’ cardiovascular risk stratification [9].

No significant differences in LDL-c concentrations were observed following the study interventions, regardless of treatment group. Systematic reviews with and without meta-analyses assessing the effects of cholesterol-lowering diets—with or without dietary supplementation—on LDL-c concentrations in individuals with FH have yielded divergent and controversial results [7,8,30]. In general, however, they suggest benefits from the use of phytosterols, with mean LDL-c reductions ranging from 23.2 mg/dL [7] to 17.4 mg/dL [8]. Substantial heterogeneity exists among the primary studies included in these reviews, particularly regarding comparison groups, study populations (children and adults, on or off lipid-lowering medication), and clinical trial designs (crossover vs. parallel). A positive impact of the DICA-FH intervention on Lp(a) concentrations was observed after follow-up, aligning with findings from other studies evaluating plant-based dietary patterns [31]. However, studies examining the effects of dietary interventions on Lp(a) concentrations remain scarce, controversial, and often contradict LDL-c-lowering recommendations [32]—particularly regarding reduced saturated fat intake [33] and the adoption of a Mediterranean diet rich in monounsaturated fatty acids [34], both of which appear to increase Lp(a) levels. In any case, our results cannot be considered definitive, as they are preliminary due to the small sample size.

Although the observed reductions were not statistically significant, there was considerable variability in both LDL-c and Lp(a) values after follow-up, reflecting the study’s small sample size but also suggesting substantial interindividual variability in response to the interventions. Given that genetic variations can modulate the metabolism of phytosterols and fatty acids, as well as their associations with different phenotypes [35,36], even under similar dietary conditions, a pharmacogenomic analysis in this FH population could help clarify this variability. Furthermore, considering that the complex and controversial relationship between diet, LDL-c, and Lp(a) remains poorly understood—particularly in the context of FH, which also involves a significant polygenic component [37]—the future individualization of dietary treatment in these individuals, based on baseline biomarkers and potentially their genotypic profile, should be considered.

The implementation of a larger-scale clinical trial appears feasible based on adherence metrics proposed in this pilot study, such as attendance at follow-up visits and capsule intake of investigational products. However, improvements in overall diet quality were modest and not statistically significant. In this regard, two key considerations should be addressed in the planning and execution of a future trial: (1) the reassessment of the dietary data collection tool, as adherence to the decentralized data collection strategy was very low, limiting dietary assessment to the CHDI method; and (2) the review of the DICA-FH strategy, as the qualitative dietary guidance provided by researchers may not have been sufficient to engage participants or emphasize the importance of incorporating Portfolio Diet foods. Additionally, investigating barriers to adherence to non-pharmacological treatment in the context of FH would be an important component to incorporate into a larger national clinical trial, as highlighted by findings from this pilot study.

The DICA-FH intervention differentially modulated plasma phytosterol biomarkers (including their corrections for total cholesterol and LDL-c concentrations, which appear to more accurately reflect plasma levels [38]) as well as erythrocyte fatty acid profiles. In the active phytosterol group, although the changes were modest and not statistically significant, there was a general increase in plasma phytosterol concentrations compared to the placebo group. Given that both groups were encouraged to consume plant-based foods—which are natural sources of these compounds [39]—the dietary component itself may have masked the effects of supplementation. Participants in the active krill oil group showed an increase in the erythrocyte omega-3/omega-6 ratio compared to the placebo group; a similar intra-group effect was observed in the active phytosterol group, likely due to a higher reported intake of fish at the end of the study. Although higher erythrocyte n-3 PUFA concentrations are associated with lower overall cardiovascular risk [40] and reduced subclinical atherosclerosis burden [41] in the general population, the clinical significance of these biomarkers remains unclear in the context of FH. In our study, they were primarily used as nutrient-specific adherence markers for the supplementation protocols. Moreover, these analyses are exploratory and should not be interpreted as definitive.

Although the dropout rates observed in this pilot study are deemed acceptable in the context of nutrition-related research [42], this indicator requires reassessment for the implementation of a larger-scale clinical trial, as the observed rate was slightly higher than the value anticipated in the study protocol [18]. The recruitment rate observed in this pilot study (approximately 15 participants/month) will also serve as a basis for recalculating the number of participating centers in a potential larger multicenter study, as well as for estimating the time required to complete participant enrollment.

This study has limitations. A slight imbalance between groups was observed in terms of the number of participants randomized to each group, which is attributable to random chance and the small sample size. Although the number of participants lost to follow-up was low in each group, it had a proportionally significant impact due to the limited sample size in each group. No statistical significance was found in the comparisons between groups concerning the primary outcomes, which is methodologically acceptable in pilot clinical trials. Furthermore, corrections for multiple testing were not performed, which may result in Type I errors in the findings. We did not collect data related to medical geography or pollution-related factors, which could also have had an impact on our outcomes. Among the strengths of this study are the following: the implementation of biochemical analyses that are underexplored in the context of dietary interventions in FH; and the potential for improvements in the event of a larger clinical trial to ensure internal validity and the generalizability of the results to our target population, such as enhancements in the development of the DICA-FH intervention, trial methodology (including the randomization strategy with unique coding, as there was only one serious adverse event, which was unrelated to the intervention), logistics, recruitment and adherence strategies, and sample size calculation.

## 5. Conclusions

In conclusion, this study demonstrated preliminary results regarding the impact of the DICA-FH intervention, with or without supplementation of phytosterols and/or krill oil, on various parameters relevant to FH such Lp(a), LDL-ox and diet quality, as well as the safety of the interventions. More importantly, the results of this pilot trial showed the feasibility of conducting a larger multicenter study in Brazil, providing valuable insights for improving the organization of such a study. Due to its limitations and the fact that this is a pilot study, our results should be interpreted with caution. If conducted, a larger study could identify new dietary treatment strategies for individuals with FH.

## Figures and Tables

**Table 1:** (**a**) Baseline characteristics of participants allocated to the phytosterol group, including both active and placebo subgroups. (**b**) Baseline characteristics of participants allocated to the krill oil group, including both active and placebo subgroups.

(a)		
	Phytosterol Placebo (*n* = 31)	Active Phytosterol (*n* = 27)
Sex, no./total no. (%)		
Female	18/31 (58.1%)	16/27 (59.3%)
Male	13/31 (41.9%)	11/27 (40.7%)
Age, in years, mean (SD)	53.8 (14.1)	55.3 (13.4)
Race		
White	17/31 (54.8%)	17/27 (63%)
Pardo	8/31 (25.8%)	5/27 (18.5%)
Other	6/31 (19.4%)	5/27 (18.5%)
Marital status, no./total no. (%)		
Married	19/31 (61.3%)	15/27 (55.6%)
Other	12/31 (38.7%)	12/27 (44.4%)
Average monthly household income, in USD ^1^, no./total no. (%)		
≥1231.17	20/31 (64.5%)	17/27 (63%)
<1231.17	11/31 (34.5%)	10/27 (37%)
Education level, in years of schooling, no./total no. (%)		
≤9 years	9/31 (29%)	9/27 (33%)
>9 years	22/31 (71%)	18/27 (67%)
Physical activity, no./total no. (%)		
Sedentary/Low	18/31 (58.1%)	15/27 (55.6%)
Moderate	10/31 (32.3%)	7/27 (25.9%)
High	3/31 (9.7%)	5/27 (18.5%)
Family history of FH, no./total no. (%)		
Unknown	3/31 (9.7%)	5/27 (18.5%)
No	5/31 (16.1%)	4/27 (14.8%)
Yes	23/31 (74.2%)	18/27 (66.7%)
Family history of premature CAD, no./total no. (%)		
Unknown	2/31 (6.5%)	1/27 (3.7%)
No	11/31 (35.5%)	8/27 (29.6%)
Yes	18/31 (58.1%)	18/27 (66.7%)
Premature CAD in a first-degree relative, no./total no. (%)		
Male < 55 years	14/18 (77.8%)	12/18 (66.7%)
Female < 65 years	4/18 (22.2%)	6/18 (33.3%)
Time since FH diagnosis, in years, median [quartiles]	2 [1–7.8]	7 [0.5–12.5]
Current smoking, no./total no. (%)	1/31 (3.2%)	4/27 (14.8%)
Alcohol consumption, no./total no. (%)	10/31 (32.3%)	16/27 (59.3%)
Estimated daily ethanol consumption, in g, median [quartiles]	2.3 [2.1–7] (*n* = 10)	2.4 [1.2–3.9] (*n* = 16)
Type 2 Diabetes, no./total no. (%)	9/31 (29%)	10/27 (37%)
Hypertension, no./total no. (%)	22/31 (71%)	22/27 (81.5%)
Previous cardiovascular events, no./total no. (%)		
No	15/31 (48.4%)	13/27 (48.1%)
Yes	16/31 (51.6%)	14/27 (51.9%)
Myocardial Infarction, no./total no. (%)	10/16 (62.5%)	10/14 (71.4%)
Stroke, no./total no. (%)	2/16 (12.5%)	0/14 (0%)
Other, no./total no. (%)	4/16 (25%)	4/14 (28.6%)
Body weight, in kg, mean (SD)	79.9 (16)	77.6 (14.8)
Body mass index, in kg/m^2^, mean (SD)	29.3 (4.1)	29.5 (4.3)
Waist circumference, in cm, mean (SD)	96.8 (11.2)	96.1 (13.9)
Dutch MEDPED, in points, mean (SD)	9.3 (3.6) (*n* = 31)	8.4 (3.6) (*n* = 26)
FH diagnosis according to Dutch MEDPED, no./total no. (%)		
Probable (6–8 points)	17/31 (54.8%)	19/26 (73.1%)
Definitive (>8 points)	14/31 (45.2%)	7/26 (26.9%)
(**b**)		
	**Krill Oil Placebo (*n* = 29)**	**Krill Oil (*n* = 29)**
Sex, no./total no. (%)		
Female	13/29 (44.8%)	21/29 (72.4%)
Male	16/29 (55.2%)	8/29 (27.6%)
Age, in years, mean (SD)	52.6 (13.4)	56.4 (14)
Race		
White	19/29 (65.5%)	15/29 (51.7%)
Pardo	5/29 (17.2%)	8/29 (27.6%)
Other	5/29 (17.3%)	6/29 (20.7%)
Marital status, no./total no. (%)		
Married	18/29 (62.1%)	16/29 (55.2%)
Other	11/29 (37.9%)	13/29 (44.8%)
Average monthly household income, in USD ^1^, no./total no. (%)		
≥1231.17	17/29 (58.6%)	20/29 (69%)
<1231.17	12/29 (41.4%)	9/29 (31%)
Education level, in years of schooling, no./total no. (%)		
≤9 years	9/29 (69%)	9/29 (69%)
>9 years	20/29 (31%)	20/29 (31%)
Physical activity, no./total no. (%)		
Sedentary/Low	15/29 (51.7%)	18/29 (62.1%)
Moderate	11/29 (37.9%)	6/29 (20.7%)
High	3/29 (10.3%)	5/29 (17.2%)
Family history of FH, no./total no. (%)		
Unknown	3/29 (10.3%)	5/29 (17.2%)
No	4/29 (13.8%)	5/29 (17.2%)
Yes	22/29 (75.9%)	19/29 (65.5%)
Family history of premature CAD, no./total no. (%)		
Unknown	1/29 (3.4%)	2/29 (6.9%)
No	9/29 (31%)	10/29 (34.5%)
Yes	19/29 (65.5%)	17/29 (58.6%)
Premature CAD in a first-degree relative, no./total no. (%)		
Male < 55 years	13/19 (68.4%)	13/17 (76.5%)
Female < 65 years	6/19 (31.6%)	4/17 (23.5%)
Time since FH diagnosis, in years, median [quartiles]	6 [1–11]	2 [0–14]
Current smoking, no./total no. (%)	3/29 (10.3%)	2/29 (6.9%)
Alcohol consumption, no./total no. (%)	15/29 (51.7%)	11/29 (37.9%)
Estimated daily ethanol consumption, in g, median [quartiles]	2.5 [1.9–6.7] (*n* = 15)	2.1 [1.6–3.5] (*n* = 11)
Type 2 Diabetes, no./total no. (%)	12/29 (41.4%)	7/29 (24.1%)
Hypertension, no./total no. (%)	23/29 (79.3%)	21/29 (72.4%)
Previous cardiovascular events, no./total no. (%)		
No	13/29 (44.8%)	15/29 (51.7%)
Yes	16/29 (55.2%)	14/29 (48.3%)
Myocardial Infarction, no./total no. (%)	11/16 (68.8%)	9/14 (64.3%)
Stroke, no./total no. (%)	1/16 (6.2%)	1/14 (7.1%)
Other, no./total no. (%)	4/16 (25%)	4/14 (28.6%)
Body weight, in kg, mean (SD)	78 (16.1)	79.7 (14.9)
Body mass index, in kg/m^2^, mean (SD)	28.5 (4.5)	30.3 (3.5)
Waist circumference, in cm, mean (SD)	95.6 (12.8)	97.4 (12.1)
Dutch MEDPED, in points, mean (SD)	8.9 (3.2) (*n* = 28)	8.9 (2.8) (*n* = 29)
FH diagnosis according to Dutch MEDPED, no./total no. (%)		
Probable (6–8 points)	18/29 (62.1%)	18/28 (64.3%)
Definitive (>8 points)	11/29 (37.9%)	10/28 (35.7%)

^1^ USD 1 = 5.70 Brazilian Reais. SD: standard deviation; CAD: coronary artery disease; FH: familial hypercholesterolemia.

**Table 2:** Comparisons of LDL-c and Lp(a) concentrations within and between study groups, before and after the intervention.

	Phytosterol			Krill Oil		
	Placebo (*n* = 26)	Active (*n* = 23)	Difference 95%CI ^1^	Placebo (*n* = 25)	Active (*n =* 24)	Difference 95% CI ^1^
LDL-c, mg/dL						
Baseline	132.5 [108.9–151.6]	119.6 [108.8–151.6]	−7.6 (−32.4; 21.1)	114.4 [81.4–141.1]	137.2 [114.9–158]	27.4 (3.1; 54)
Final	119.2 [92.9–148.7]	133.3 [90.8–162.8]	0.1 (−36.6; 32.9)	112.6 [77.94–161.93]	120.9 [100.7–164.5]	15 (−18.2; 49.2)
Difference, in mg/dL (95%CI) ^2^	−8.2 (−18.3; 6.3)	0.6 (−14.4; 20.1)	6.2 (−10.6; 26.3)	1 (−10.3; 14.6)	−11.9 (−23.2; 15.6)	−12.1 (−28; 4.5)
Difference, in % (IQR) ^3^	−5.9 [−17.4–1.7]	0 [−18.31–22.85]	3.3 (−12.9; 20.9)	−1.9 [−10.7–19.6]	−11.3 [−21.7–1.4]	−8.1 (−22.3; 6.8)
Lp(a), mg/dL						
Baseline	27.7 [20.3–55.5]	38.1 [15.2–64]	1.2 (−16; 21.7)	35.3 [16.1–57.1]	30.6 [18.9–63.6]	1.2 (−17; 17.8)
Final	27.4 [19.2–50.8]	25.8 [13.5–74.6]	−0.9 (−15.8; 17.1)	23.4 [14.4–51.3]	31.2 [17.6–56.9]	3.3 (−10; 19.3)
Difference, in mg (95%CI) ^2^	−3 (−7.2; −0.3) *	−5.2 (−14.6; 0.2)	−1.00 (−7.00; 3.30)	−5.8 (−10.5; −1.5) ^¶^	−2.3 (−7.9; 2.2)	1.7 (−2.5; 8.2)
Difference, in % (IQR) ^3^	−10.9 [−21.6–1.9] *	−8.9 [−25.2–0]	−1.6 (−12.4; 12.4)	−16.1 [−25.2–−0.8] ^¶^	−8.1 [−18.4–5.8]	8.2 (−3.4; 21.6)

^1^ Difference in medians between groups (active–placebo) using the Hodges–Lehmann estimator. ^2^ Intra-group median difference by the paired Wilcoxon test; 95% confidence interval (95% CI) estimated for the median of the differences between paired observations. ^3^ Percentual difference in intra-group medians, calculated as: 100 × (final − initial)/final. * *p* = 0.03; ^¶^ *p* < 0.001. LDL-c: low-density lipoprotein cholesterol; Lp(a): lipoprotein(a); IQR: interquartile range.

**Table 3:** Comparisons of the Cardiovascular Health Diet Index (individual components and total points) within and between study groups, before and after the intervention.

	Phytosterol			Krill Oil		
	Placebo	Active	Difference 95%CI ^1^	Placebo	Active	Difference 95%CI ^1^
Fruits, points						
Baseline	6.2 ± 3.9 (*n* = 31)	6.7 ± 3.7 (*n* = 27)	0.47 (−1.50; 2.50)	6.5 ± 3.9 (*n* = 29)	6.4 ± 3.8 (*n* = 29)	−0.07 (−2.10; 1.90)
Final	7.7 ± 3.2 (*n* = 26)	7.1 ± 3.9 (*n* = 24)	−0.61 (−2.65; 1.43)	7.6 ± 3.6 (*n* = 25)	7.2 ± 3.6 (*n* = 25)	−0.40 (−2.43; 1.63)
Vegetables, points						
Baseline	2.2 ± 1.6 (*n* = 31)	2.9 ± 1.9 (*n* = 27)	0.72 (−0.20; 1.60)	2.5 ± 1.7 (*n* = 29)	2.5 ± 1.9 (*n* = 29)	−0.03 (−1.00; 0.90)
Final	2.6 ± 1.9 (*n* = 26)	3.1 ± 2 (*n* = 24)	0.56 (−0.55; 1.67)	2.9 ± 1.9 (*n* = 25)	2.8 ± 2 (*n* = 25)	−0.07 (−1.18; 1.05)
Whole grains, points						
Baseline	2 ± 2.9 (*n* = 31)	3.1 ± 3.3 (*n* = 27)	1.04 (−0.60; 2.70)	3 ± 3.3 (*n* = 29)	2.1 ± 3 (*n* = 29)	−0.92 (−2.60; 0.70)
Final	3.5 ± 3.4 (*n* = 26)	3.5 ± 3.3 (*n* = 24)	−0.04 (−1.96; 1.88)	3.8 ± 3.5 (*n* = 25)	3.2 ± 3.3 (*n* = 25)	−0.59 (−2.50; 1.32)
Legumes, points						
Baseline	8.4 ± 3.7 (*n* = 31)	8.5 ± 3.6 (*n* = 27)	0.13 (−1.80; 2.10)	8.6 ± 3.5 (*n* = 29)	8.3 ± 3.8 (*n* = 29)	−0.34 (−2.30; 1.60)
Final	9.2 ± 2.7 (*n* = 26)	9.6 ± 2 (*n* = 24)	0.35 (−1.01; 1.71)	9.6 ± 2 (*n* = 25)	9.2 ± 2.8 (*n* = 25)	−0.40 (−1.78; 0.98)
Nuts, points						
Baseline	3.1 ± 4.4 (*n* = 31)	4.4 ± 4.4 (*n* = 27)	1.33 (−1.00; 3.70)	3.2 ± 4.4 (*n* = 29)	4.3 ± 4.4 (*n* = 29)	1.03 (−1.30; 3.40)
Final	4.2 ± 4.1 (*n* = 26)	4.5 ± 4.4 (*n* = 24)	0.28 (−2.14; 2.70)	4.2 ± 3.9 (*n* = 25)	4.5 ± 4.5 (*n* = 25)	0.33 (−2.07; 2.74)
Dairy, points						
Baseline	4.8 ± 5.1 (*n* = 31)	4.1 ± 5 (*n* = 27)	−0.76 (−3.40; 1.90)	4.5 ± 5.1 (*n* = 29)	4.5 ± 5.1 (*n* = 29)	0.00 (−2.70; 2.70)
Final	5.8 ± 5 (*n* = 26)	5.4 ± 5.1 (*n* = 24)	−0.35 (−3.24; 2.53)	6 ± 5 (*n* = 25)	5.2 ± 5.1 (*n* = 25)	−0.80 (−3.67; 2.07)
Fish and seafood, points						
Baseline	1.8 ± 2.8 (*n* = 31)	3.5 ± 3.6 (*n* = 27)	1.74 (0.00; 3.50)	3.3 ± 3.6 (*n* = 29)	1.9 ± 2.8 (*n* = 29)	−1.38 (−3.10; 0.30)
Final	3.1 ± 3.5 (*n* = 26)	5.4 ± 4.4 (*n* = 24)	2.34 (0.06; 4.62) *	4.2 ± 4.5 (*n* = 25)	4.2 ± 3.7 (*n* = 25)	0.00 (−2.35; 2.35)
Red meat, points						
Baseline	3.2 ± 4.8 (*n* = 31)	1.9 ± 4 (*n* = 27)	−1.37 (−3.70; 0.90)	2.4 ± 4.4 (*n* = 29)	2.8 ± 4.5 (*n* = 29)	0.34 (−2.00; 2.70)
Final	2.7 ± 4.5 (*n* = 26)	2.1 ± 4.1 (*n* = 24)	−0.61 (−3.07; 1.86)	2 ± 4.1 (*n* = 25)	2.8 ± 4.6 (*n* = 25)	0.80 (−1.67; 3.27)
Processed meat, points						
Baseline	9.4 ± 2.5 (*n* = 31)	9.3 ± 2.7 (*n* = 27)	−0.10 (−1.50; 1.30)	9.3 ± 2.6 (*n* = 29)	9.3 ± 2.6 (*n* = 29)	0.00 (−1.40; 1.40)
Final	10 ± 0 (*n* = 26)	8.8 ± 3.4 (*n* = 24)	−1.25 (−2.68; 0.18)	9.2 ± 2.8 (*n* = 25)	9.6 ± 2 (*n* = 25)	0.40 (−0.98; 1.78)
Sweet sugar beverages, points						
Baseline	6.5 ± 4.9 (*n* = 31)	7.8 ± 4.2 (*n* = 27)	1.33 (−1.10; 3.70)	6.2 ± 4.9 (*n* = 29)	7.9 ± 4.1 (*n* = 29)	1.72 (−0.70; 4.10)
Final	7.7 ± 4.3 (*n* = 26)	7.1 ± 4.6 (*n* = 24)	−0.61 (−3.16; 1.94)	8 ± 4.1 (*n* = 25)	6.8 ± 4.8 (*n* = 25)	−1.20 (−3.72; 1.32)
Ultra-processed food, points						
Baseline	10 ± 0 (*n* = 31)	10 ± 0 (*n* = 27)	-	10 ± 0 (*n* = 29)	10 ± 0 (*n* = 29)	-
Final	10 ± 0 (*n* = 26)	10 ± 0 (*n* = 24)	-	10 ± 0 (*n* = 25)	10 ± 0 (*n* = 25)	-
CHDI, total points						
Baseline	57.5 ± 16.5 (*n* = 31)	62.1 ± 15.6 (*n* = 27)	4.53 (−3.90; 13.00)	59.5 ± 16.2 (*n* = 29)	59.8 ± 16.3 (*n* = 29)	0.36 (−8.20; 8.90)
Final	66.5 ± 14.7 (*n* = 26)	66.5 ± 15.3 (*n* = 24)	0.06 (−8.49; 8.62)	67.5 ± 16 (*n* = 25)	65.5 ± 13.9 (*n* = 25)	−1.92 (−10.44; 6.60)

^1^ Difference in means between groups (active—placebo) using Student’s *t*-test. * *p* = 0.04. CHDI: Cardiovascular Health Diet Index.

**Table 4:** Comparisons of the plasma phytosterol concentrations (adjusted or not for TC and LDL-c) within and between study groups, before and after the intervention.

	Phytosterol			Krill Oil		
	Placebo (*n* = 20)	Active (*n* = 20)	Difference 95%CI ^1^	Placebo (*n* = 20)	Active (*n* = 20)	Difference 95%CI ^1^
**Campesterol, µg/mL**						
Baseline	0.44 [0.13–0.71]	0.36 [0.18–0.52]	−0.03 (−0.31; 0.17)	0.36 [0.18–0.65]	0.41 [0.14–0.71]	0.01 (−0.21; 0.25)
Final	0.14 [0.09–0.57]	0.43 [0.13–0.79]	0.09 (−0.03; 0.49)	0.21 [0.09–0.61]	0.25 [0.12–0.74]	0.04 (−0.09; 0.34)
Difference, in µg/mL (95%CI) ^2^	−0.17 (−0.59; 0.09)	0.09 (−0.2; 0.52)	0.32 (−0.15; 0.76)	−0.12 (−0.4; 0.14)	0.02 (−0.33; 0.47)	0.15 (−0.32; 0.62)
**Campesterol, µg/mg TC ^3^**						
Baseline	0.21 [0.05–0.39]	0.17 [0.08–0.31]	0.00 (−0.15; 0.08)	0.17 [0.08–0.36]	0.21 [0.07–0.34]	−0.01 (−0.14; 0.10)
Final	0.08 [0.05–0.32]	0.12 [0.07–0.44]	0.05 (−0.02; 0.19)	0.12 [0.05–0.31]	0.11 [0.06–0.35]	0.01 (−0.07; 0.11)
Difference, in µg/mg TC (95%CI) ^2^	−0.08 (−0.23; 0.03)	0.04 (−0.11; 0.28)	0.14 (−0.08; 0.36)	−0.05 (−0.21; 0.07)	0.02 (−0.14; 0.25)	0.09 (−0.14; 0.31)
**Campesterol, µg/mg LDL-c ^3^**						
Baseline	0.34 [0.07–0.61]	0.25 [0.12–0.48]	0.03 (−0.25; 0.17)	0.28 [0.11–0.64]	0.27 [0.09–0.5]	−0.04 (−0.27; 0.13)
Final	0.14 [0.08–0.48]	0.33 [0.12–0.7]	0.07 (−0.05; 0.32)	0.24 [0.09–0.48]	0.18 [0.09–0.58]	0.01 (−0.19; 0.18)
Difference, in µg/mg LDL-c (95%CI) ^2^	−0.12 (−0.33; 0.07)	0.10 (−0.19; 0.53)	0.20 (−0.12; 0.61)	−0.08 (−0.34; 0.1)	0.04 (−0.2; 0.38)	0.16 (−0.21; 0.54)
**Stigmasterol, µg/mL**						
Baseline	0.28 [0.14–0.52]	0.1 [0.03–0.19]	−0.16 (−0.32; −0.05) *	0.24 [0.1–0.36]	0.15 [0.06–0.26]	−0.06 (−0.20; 0.06)
Final	0.05 [0.02–0.15]	0.13 [0.05–0.27]	0.05 (−0.01; 0.13)	0.09 [0.04–0.18]	0.08 [0.02–0.3]	0.00 (−0.06; 0.11)
Difference, in µg/mL (95%CI) ^2^	−0.24 (−0.6; −0.1) *	0.03 (−0.06; 0.15)	0.28 (0.10; 0.48) *	−0.16 (−0.41; −0.03) *	−0.01 (−0.16; 0.11)	0.14 (−0.04; 0.35)
**Stigmasterol, µg/mg TC ^3^**						
Baseline	0.13 [0.07–0.25]	0.06 [0.02–0.09]	−0.07 (−0.17; −0.01) *	0.08 [0.05–0.22]	0.07 [0.03–0.12]	−0.03 (−0.09; 0.02)
Final	0.03 [0.01–0.1]	0.08 [0.03–0.11]	0.02 (−0.01; 0.06)	0.06 [0.02–0.1]	0.04 [0.01–0.11]	−0.01 (−0.04; 0.03)
Difference, in µg/mg TC (95%CI) ^2^	−0.11 (−0.3; −0.05) *	0.01 (−0.03; 0.07)	0.12 (0.05; 0.22) *	−0.08 (−0.2; −0.01) ^¶^	−0.01 (−0.08; 0.05)	0.05 (−0.03; 0.15)
**Stigmasterol, µg/mg LDL-c ^3^**						
Baseline	0.23 [0.09–0.39]	0.1 [0.03–0.16]	−0.11 (−0.27; −0.01) ^2^	0.15 [0.09–0.34]	0.12 [0.03–0.23]	−0.05 (−0.16; 0.04)
Final	0.05 [0.02–0.15]	0.14 [0.06–0.19]	0.05 (−0.02; 0.12)	0.1 [0.04–0.17]	0.06 [0.02–0.17]	−0.01 (−0.08; 0.04)
Difference, in µg/mg LDL-c (95%CI) ^2^	−0.18 (−0.53; −0.07) *	0.04 (−0.05; 0.13)	0.20 (0.08; 0.39) *	−0.11 (−0.3; −0.004) ^≠^	−0.02 (−0.13; 0.09)	0.10 (−0.05; 0.26)
**Beta-sitosterol, µg/mL**						
Baseline	0.2 [0.15–1.84]	0.37 [0.1–1.7]	−0.04 (−0.19; 0.69)	0.23 [0.12–1.89]	0.2 [0.12–1.65]	−0.01 (−0.65; 0.14)
Final	0.04 [0.02–0.1]	0.07 [0.02–0.15]	0.01 (−0.03; 0.07)	0.07 [0.02–0.12]	0.06 [0.02–0.14]	0.00 (−0.04; 0.05)
Difference, in µg/mL (95%CI) ^2^	−0.81 (−1.25; −0.13) *	−0.79 (−1.47; −0.13) *	0.04 (−0.33; 0.31)	−0.89 (−1.67; −0.1) *	−0.64 (−1.23; −0.13) *	−0.01 (−0.21; 0.63)
**Beta-sitosterol, µg/mg TC ^3^**						
Baseline	0.1 [0.06–0.84]	0.12 [0.05–0.81]	−0.01 (−0.08; 0.40)	0.1 [0.06–0.99]	0.11 [0.06–0.71]	0.00 (−0.42; 0.06)
Final	0.02 [0.01–0.05]	0.04 [0.01–0.07]	0.00 (−0.01; 0.03)	0.03 [0.01–0.07]	0.03 [0.01–0.05]	0.00 (−0.03; 0.02)
Difference, in µg/mg TC (95%CI) ^2^	−0.35 (−0.65; −0.05) *	−0.37 (−0.76; −0.05) *	0.00 (−0.16; 0.11)	−0.44 (−0.78; −0.04) *	−0.24 (−0.55; −0.06) *	−0.01 (−0.08; 0.37)
**Beta-sitosterol, µg/mg LDL-c ^3^**						
Baseline	0.16 [0.09–1.23]	0.24 [0.1–1.47]	0.01 (−0.11; 0.81)	0.2 [0.09–1.68]	0.16 [0.1–1.27]	−0.02 (−0.58; 0.10)
Final	0.04 [0.02–0.08]	0.06 [0.03–0.12]	0.01 (−0.02; 0.06)	0.06 [0.02–0.12]	0.05 [0.02–0.09]	0.00 (−0.05; 0.03)
Difference, in µg/mg LDL-c (95%CI) ^2^	−0.49 (−1.04; −0.08) *	−0.71 (−1.13; −0.08) *	−0.02 (−0.48; 0.25)	−0.74 (−1.46; −0.08)	−0.42 (−0.92; −0.09)	−0.01 (−0.20; 0.62)

^1^ Difference in medians between groups (active—placebo) using the Hodges–Lehmann estimator. ^2^ Intra-group median difference by the paired Wilcoxon test; 95% confidence interval (95% CI) estimated for the median of the differences between paired observations. ^3^ Corrections for cholesterol were calculated using the ratio of sterol content (in µg) to cholesterol levels (either TC or LDL-c, in mg). TC: total cholesterol; LDL-c: low-density lipoprotein cholesterol. * *p* = < 0.01; ^¶^ *p* = 0.02; ≠ *p* = 0.04.

**Table 5:** Comparisons of omega-3 polyunsaturated fatty acids biomarkers within and between study groups, before and after the intervention.

	Phytosterol			Krill Oil		
	Placebo (*n* = 24)	Active (*n* = 23)	Difference 95%CI ^1^	Placebo (*n* = 24)	Active (*n* = 23)	Difference 95%CI ^1^
**(EPA) Eicosapentaenoic (C20:5 n3), %**						
Baseline	0.46 [0.39–0.62]	0.49 [0.41–0.6]	0.02 (−0.09; 0.12)	0.46 [0.39–0.6]	0.49 [0.36–0.65]	0.00 (−0.11; 0.10)
Final	0.3 [0.02–0.57]	0.34 [0.12–0.66]	0.06 (−0.13; 0.27)	0.25 [0.03–0.37]	0.47 [0.08–0.85]	0.21 (−0.01; 0.47)
Difference (95%CI) ^2^	−0.17 (−0.39; 0.05)	−0.09 (−0.27; 0.12)	0.10 (−0.20; 0.34)	−0.24 (−0.40; −0.06) *	0.02 (−0.2; 0.24)	0.24 (−0.04; 0.52)
**(DHA) Docosahexaenoic (C22:6 n3), %**						
Baseline	3.54 ± 0.95	3.34 ± 0.97	−0.20 (−0.77; 0.36)	3.57 ± 0.77	3.32 ± 1.12	−0.25 (−0.82; 0.32)
Final	4.33 ± 1.3	4.46 ± 1.35	0.13 (−0.65; 0.91)	4.13 ± 1.33	4.67 ± 1.25	0.54 (−0.22; 1.30)
Difference (95%CI) ^2^	0.78 (0.2; 1.37) ^●^	1.12 (0.54; 1.69) *	0.33 (−0.46; 1.13)	0.56 (0.07; 1.05) ^¶^	1.35 (0.72; 1.97) *	0.79 (0.02; 1.56) ^●^
**Omega−3 Index, % ^3^**						
Baseline	4.07 [3.36–4.69]	3.6 [3.29–4.25]	−0.24 (−0.85; 0.32)	3.83 [3.35–4.85]	3.68 [3.36–4.25]	−0.15 (−0.91; 0.38)
Final	4.54 [3.64–5.37]	4.63 [3.87–5.88]	0.15 (−0.64; 1.21)	4.23 [3.07–5.07]	4.81 [4.46–5.93]	0.85 (−0.04; 1.67)
Difference (95%CI) ^2^	0.53 (−0.30; 1.40)	1.10 (0.31; 1.92) *	0.50 (−0.41; 1.54)	0.27 (−0.35; 1.03)	1.38 (0.44; 2.17) *	0.95 (0.04; 1.87)
**Omega-3/Omega-6 ratio ^4^**						
Baseline	0.15 [0.13–0.19]	0.13 [0.12–0.17]	−0.01 (−0.04; 0.01)	0.15 [0.12–0.2]	0.13 [0.12–0.16]	−0.02 (−0.05; 0.01)
Final	0.18 [0.15–0.23]	0.17 [0.15–0.24]	0.01 (−0.02; 0.05)	0.16 [0.12–0.2]	0.18 [0.17–0.24]	0.03 (−0.01; 0.07)
Difference (95%CI) ^2^	0.02 (−0.013; 0.051)	0.05 (0.03; 0.08) *	0.03 (−0.01; 0.07)	0.01 (−0.023; 0.04)	0.06 (0.03; 0.08)	0.05 (0.01; 0.09) ^●^

^1^ Difference in medians between groups (active—placebo) using the Hodges–Lehmann estimator; for docosahexaenoic (C22:6 n3) fatty acid differences in mean percentage between groups (active—placebo) using the non-paired Student’s *t*-test. ^2^ Intra-group median difference by the paired Wilcoxon test; 95% confidence interval (95% CI) estimated for the median of the differences between paired observations; for docosahexaenoic (C22:6 n3) fatty acid, intra-group mean difference by the paired *t* test; 95% CI estimated for the mean of the differences between paired observations. ^3^ Omega-3 Index assessed by the sum of eicosapentaenoic acid (EPA) (C20:5 n3) and docosahexaenoic acid (DHA) (C22:6 n3). ^4^ Omega-3/Omega-6 ratio calculated as: cis-11,14,17 eicosatrienoic acid (C20:3) + eicosapentaenoic acid (EPA) (C20:5) + docosahexaenoic acid (DHA) (C22:6) divided by linoleic acid (C18:2) + linoelaidic acid (C18:2) + cis-8,11,14 eicosatrienoic acid (C20:3) + arachidonic acid (C20:4) + cis-11,14-eicosadienoic acid (C20:2). ^¶^ *p* = 0.03; * *p* = < 0.01; ^●^ *p* = 0.01.

## Data Availability

Data and materials will be made available upon reasonable request to the corresponding author, following the completion of a specific form provided by the Hcor Research Institute and in accordance with the institution’s data sharing policies.

## Supplementary Materials

The following supporting information can be downloaded at https://www.mdpi.com/article/10.3390/nu17122008/s1, Table S1. Consolidated Standards of Reporting Trials (CONSORT) checklist for pilot and feasibility trials; Table S2. Lipid-lowering therapy in use at baseline in the phytosterol (active and placebo) intervention group; Table S3. Lipid-lowering therapy in use at baseline in the krill oil (active and placebo) intervention group; Table S4. Baseline characteristics considering the four randomization subgroups; Table S5. Lipid-lowering therapy in use at baseline in the four study subgroups; Table S6. Pathogenic genetic variants related to familial hypercholesterolemia identified in participants of the DICA-FH study; Table S7. Changes in plasma phytosterol biomarkers following the DICA-FH intervention, irrespective of study groups; Table S8. Comparisons of erythrocyte fatty acids within and between study groups, before and after the intervention; Table S9. Changes in erythrocyte fatty acids following the DICA-FH intervention, irrespective of study groups; Table S10. Comparisons of clinical lipid profile markers within and between study groups, before and after the intervention; Table S11. Comparisons of oxidized low density lipoprotein cholesterol and apolipoproteins within and between study groups, before and after the intervention; Table S12. Adverse events reported during the study in the four study subgroups; Table S13. Comparisons of liver enzymes within and between study groups, before and after the intervention.

## Abbreviations

The following abbreviations are used in this manuscript:

*ABCG8*	ATP binding cassette subfamily G member 8 gene
Apo(s)	Apolipoproteins
*APOB*	Apolipoprotein B gene
*APOE*	Apolipoprotein E gene
BMI	Body mass index
CAD	Coronary artery disease
CHDI	Cardiovascular Health Diet Index
CI	Confidence interval
CONSORT	Consolidated Standards of Reporting Trials
DHA	Docosahexaenoic acid
DICA Br	*DIeta CArdioprotetora Brasileira*
DICA-FH	*DIeta CArdioprotetora Brasileira* adapted to familial hypercholesterolemia
Dutch MEDPED	Dutch Lipid Clinic Network
eCRF	Electronic case report form
EPA	Eicosapentaenoic acid
FH	Familial hypercholesterolemia
HDL-c	High-density lipoprotein cholesterol
IQR	Interquartile range
LDL-c	Low-density lipoprotein cholesterol
*LDLR*	LDL receptor gene
Lp(a)	Lipoprotein(a)
n-3 PUFA	omega-3 polyunsaturated fatty acid
ox-LDL	Oxidized LDL
*PCSK9*	Proprotein convertase subtilisin/kexin type 9 gene
R24h	24-h dietary recall
SD	Standard deviation
*STAP1*	Signal transducing adaptor family member 1 gene
VLDL-c	Very-low density lipoprotein cholesterol
WHO-UTN	World Health Organization Universal Trial Number
