# Supplementary material for: An Adapted Cardioprotective Diet with or Without Phytosterol and/or Krill Oil Supplementation in Familial Hypercholesterolemia: Results of a Pilot Randomized Clinical Trial
Source: Nutrients. 2025 Jun 15;17(12):2008. doi: 10.3390/nu17122008 (PMC12196534; doi:10.3390/nu17122008)
Supplement: Supplementary file 1 [file nutrients-17-02008-s001.zip › nutrients-3664120-supplementary.pdf]

Supplementary Material

Table S1. Consolidated Standards of Reporting Trials (CONSORT) checklist for pilot and feasibility trials.

Section/Topic	Item No	Checklist item	Reported on page No
**Title and abstract**			
	1a	Identification as a pilot or feasibility randomised trial in the title	1
	1b	Structured summary of pilot trial design, methods, results, and conclusions (for specific guidance see CONSORT abstract extension for pilot trials)	1,2
**Introduction**			
Background and objectives	2a	Scientific background and explanation of rationale for future definitive trial, and reasons for randomised pilot trial	2,3
	2b	Specific objectives or research questions for pilot trial	3
**Methods**			
Trial design	3a	Description of pilot trial design (such as parallel, factorial) including allocation ratio	4
	3b	Important changes to methods after pilot trial commencement (such as eligibility criteria), with reasons	NA
Participants	4a	Eligibility criteria for participants	4; https://www.sciencedirect.com/science/article/pii/S2667268524000056
	4b	Settings and locations where the data were collected	4,7
	4c	How participants were identified and consented	4
Interventions	5	The interventions for each group with sufficient details to allow replication, including how and when they were actually administered	5,6; https://www.sciencedirect.com/science/article/pii/S2667268524000056

Outcomes	6a	Completely defined prespecified assessments or measurements to address each pilot trial objective specified in 2b, including how and when they were assessed	6; https://www.sciencedirect.com/science/article/pii/S2667268524000056
	6b	Any changes to pilot trial assessments or measurements after the pilot trial commenced, with reasons	NA
	6c	If applicable, prespecified criteria used to judge whether, or how, to proceed with future definitive trial	NA
Sample size	7a	Rationale for numbers in the pilot trial	6; https://www.sciencedirect.com/science/article/pii/S2667268524000056
	7b	When applicable, explanation of any interim analyses and stopping guidelines	NA
Randomisation			
Sequence generation	8a	Method used to generate the random allocation sequence	4
	8b	Type of randomisation(s); details of any restriction (such as blocking and block size)	4
Allocation concealment mechanism	9	Mechanism used to implement the random allocation sequence (such as sequentially numbered containers), describing any steps taken to conceal the sequence until interventions were assigned	4
Implementation	10	Who generated the random allocation sequence, who enrolled participants, and who assigned participants to interventions	4; https://www.sciencedirect.com/science/article/pii/S2667268524000056
Blinding	11a	If done, who was blinded after assignment to interventions (for example, participants, care providers, those assessing outcomes) and how	4; https://www.sciencedirect.com/science/article/pii/S2667268524000056

	11b	If relevant, description of the similarity of interventions	5; https://www.sciencedirect.com/science/article/pii/S2667268524000056
Statistical methods	12	Methods used to address each pilot trial objective whether qualitative or quantitative	6,7
**Results**			
Participant flow (a diagram is strongly recommended)	13a	For each group, the numbers of participants who were approached and/or assessed for eligibility, randomly assigned, received intended treatment, and were assessed for each objective	7; Flowchart
	13b	For each group, losses and exclusions after randomisation, together with reasons	7; Flowchart
Recruitment	14a	Dates defining the periods of recruitment and follow-up	4,7
	14b	Why the pilot trial ended or was stopped	NA
Baseline data	15	A table showing baseline demographic and clinical characteristics for each group	9,10, Supplementary Material
Numbers analysed	16	For each objective, number of participants (denominator) included in each analysis. If relevant, these numbers should be by randomised group	11-19; Supplementary Material
Outcomes and estimation	17	For each objective, results including expressions of uncertainty (such as 95% confidence interval) for any estimates. If relevant, these results should be by randomised group	11-19; Supplementary Material
Ancillary analyses	18	Results of any other analyses performed that could be used to inform the future definitive trial	NA
Harms	19	All important harms or unintended effects in each group (for specific guidance see CONSORT for harms)	20; Supplementary Material
	19a	If relevant, other important unintended consequences	NA
**Discussion**			
Limitations	20	Pilot trial limitations, addressing sources of potential bias and remaining uncertainty about feasibility	23
Generalisability	21	Generalisability (applicability) of pilot trial methods and findings to future definitive trial and other studies	22
Interpretation	22	Interpretation consistent with pilot trial objectives and findings, balancing potential benefits and harms, and considering other relevant evidence	21,22

	22a	Implications for progression from pilot to future definitive trial, including any proposed amendments	22,23
**Other information**			
Registration	23	Registration number for pilot trial and name of trial registry	4
Protocol	24	Where the pilot trial protocol can be accessed, if available	https://www.sciencedirect.com/science/article/pii/S2667268524000056
Funding	25	Sources of funding and other support (such as supply of drugs), role of funders	23
	26	Ethical approval or approval by research review committee, confirmed with reference number	23

Table S2. Lipid-lowering therapy in use at baseline in the phytosterol (active and placebo) intervention group.

	**Phytosterol placebo (n=31)**	**Active phytosterol (n=27)**
Atorvastatin 20 mg	1/31 (3.2%)	1/27 (3.7%)
Atorvastatin 40 mg	1/31 (3.2%)	1/27 (3.7%)
Atorvastatin 40 mg + Ezetimibe 10 mg	1/31 (3.2%)	1/27 (3.7%)
Atorvastatin 60 mg	1/31 (3.2%)	0/27 (0%)
Atorvastatin 80 mg	6/31 (19.4%)	4/27 (14.8%)
Atorvastatin 80 mg + Ezetimibe 10 mg	12/31 (38.7%)	11/27 (40.7%)
Pitavastatin 4 mg	1/31 (3.2%)	0/27 (0%)
Rosuvastatin 10 mg	1/31 (3.2%)	2/27 (7.4%)
Rosuvastatin 20 mg	0/31 (0%)	1/27 (3.7%)
Rosuvastatin 20 mg + Ezetimibe 10 mg	1/31 (3.2%)	2/27 (7.4%)
Rosuvastatin 40 mg	1/31 (3.2%)	0/27 (0%)
Rosuvastatin 40 mg + Ezetimibe 10 mg	2/31 (6.5%)	3/27 (11.1%)
Simvastatin 40 mg	3/31 (9.7%)	0/27 (0%)
Other	0/31 (0%)	1/27 (3.7%)

Table S3. Lipid-lowering therapy in use at baseline in the krill oil (active and placebo) intervention group.

	**Krill oil placebo (n=31)**	**Active krill oil (n=27)**
Atorvastatin 20 mg	0/29 (0%)	2/29 (6.9%)
Atorvastatin 40 mg	0/29 (0%)	2/29 (6.9%)
Atorvastatin 40 mg + Ezetimibe 10 mg	1/29 (3.4%)	1/29 (3.4%)
Atorvastatin 60 mg	0/29 (0%)	1/29 (3.4%)
Atorvastatin 80 mg	5/29 (17.2%)	5/29 (17.2%)
Atorvastatin 80 mg + Ezetimibe 10 mg	13/29 (44.8%)	10/29 (34.5%)
Pitavastatin 4 mg	0/29 (0%)	1/29 (3.4%)
Rosuvastatin 10 mg	2/29 (6.9%)	1/29 (3.4%)
Rosuvastatin 20 mg	0/29 (0%)	1/29 (3.4%)
Rosuvastatin 20 mg + Ezetimibe 10 mg	1/29 (3.4%)	2/29 (6.9%)
Rosuvastatin 40 mg	1/29 (3.4%)	0/29 (0%)
Rosuvastatin 40 mg + Ezetimibe 10 mg	4/29 (13.8%)	1/29 (3.4%)
Simvastatin 40 mg	1/29 (3.4%)	2/29 (6.9%)
Other	1/29 (3.4%)	0/29 (0%)

Table S4. Baseline characteristics considering the four randomization subgroups.

	Control group (n=16)	PP/KO (n=15)	P/PK (n=13)	P/KO (n=14)
Sex				
Female	7/16 (43.8%)	11/15 (73.3%)	6/13 (46.2%)	10/14 (71.4%)
Male	9/16 (56.2%)	4/15 (26.7%)	7/13 (53.8%)	4/14 (28.6%)
Age, in years, mean ± SD	50.6 ± 13	57.1 ± 14.9	54.9 ± 14	55.6 ± 13.4
Race				
White	9/16 (56.2%)	8/15 (53.3%)	10/13 (76.9%)	7/14 (50%)
Pardo	4/16 (25%)	4/15 (26.7%)	1/13 (7.7%)	4/14 (28.6%)
Other	3/16 (18.8%)	3/15 (20%)	2/13 (15.4%)	3/14 (21.4%)
Marital status				
Married	10/16 (62.5%)	9/15 (60%)	8/13 (61.5%)	7/14 (50%)
Other	6/16 (37.5%)	6/15 (40%)	5/13 (38.5%)	7/14 (50%)
Average monthly household income, in US$[1]				
≥ 1,231.17	10/16 (62.5%)	10/15 (66.7%)	7/13 (53.8%)	10/14 (71.4%)
< 1,231.17	6/16 (37.5%)	5/15 (33.3%)	6/13 (46.2%)	4/14 (28.6%)
Education level				
≤ 8 years	5/16 (6.2%)	4/15 (13.3%)	4/13 (0%)	5/14 (14.3%)
> 8 years	11/16 (25%)	11/15 (53.3%)	9/13 (23.1%)	9/14 (28.6%)
Physical activity				
Sedentary/Low	9/16 (56.2%)	9/15 (60%)	6/13 (46.2%)	9/14 (64.3%)
Moderate	6/16 (37.5%)	4/15 (26.7%)	5/13 (38.5%)	2/14 (14.3%)
High	1/16 (6.2%)	2/15 (13.3%)	2/13 (15.4%)	3/14 (21.4%)
Family history of FH				
Unknown	0/16 (0%)	3/15 (20%)	3/13 (23.1%)	2/14 (14.3%)
No	4/16 (25%)	1/15 (6.7%)	0/13 (0%)	4/14 (28.6%)
Yes	12/16 (75%)	11/15 (73.3%)	10/13 (76.9%)	8/14 (57.1%)
Family history of premature coronary artery disease				
Unknown	1/16 (6.2%)	1/15 (6.7%)	0/13 (0%)	1/14 (7.1%)

No	5/16 (31.2%)	6/15 (40%)	4/13 (30.8%)	4/14 (28.6%)
Yes	10/16 (62.5%)	8/15 (53.3%)	9/13 (69.2%)	9/14 (64.3%)
In a first-degree relative				
Male < 55 years	9/10 (90%)	5/8 (62.5%)	4/9 (44.4%)	8/9 (88.9%)
Female < 65 years	1/10 (10%)	3/8 (37.5%)	5/9 (55.6%)	1/9 (11.1%)
Time since FH diagnosis; in years, median [quartiles]	4.5 [1 - 8.8]	2 [0.2 - 6.8]	6 [1 - 11]	7.5 [0.2 - 17]
Current smoking	0/16 (0%)	1/15 (6.7%)	3/13 (23.1%)	1/14 (7.1%)
Alcohol consumption	7/16 (43.8%)	3/15 (20%)	8/13 (61.5%)	8/14 (57.1%)
Estimated daily ethanol consumption (g), median [quartiles]	2.5 [2.1 - 6.7] (n=7)	2.1 [1.3 - 5.1] (n=3)	2.7 [1 - 5.1] (n=8)	2.4 [1.7 - 3.4] (n=8)
Type 2 Diabetes	7/16 (43.8%)	2/15 (13.3%)	5/13 (38.5%)	5/14 (35.7%)
Hypertension	11/16 (68.8%)	11/15 (73.3%)	12/13 (92.3%)	10/14 (71.4%)
Previous cardiovascular events				
No	7/16 (43.8%)	8/15 (53.3%)	6/13 (46.2%)	7/14 (50%)
Yes	9/16 (56.2%)	7/15 (46.7%)	7/13 (53.8%)	7/14 (50%)
Acute Myocardial Infarction	6/9 (66.7%)	4/7 (57.1%)	5/7 (71.4%)	5/7 (71.4%)
Stroke	1/9 (11.1%)	1/7 (14.3%)	0/7 (0%)	0/7 (0%)
Other	2/9 (22.2%)	2/7 (28.6%)	2/7 (28.6%)	2/7 (28.6%)
Body weight, in kg, mean ± SD	78.1 ± 16.7	81.8 ± 15.6	77.8 ± 15.9	77.4 ± 14.4
Body mass index, in Kg/m2, mean ± SD	28.1 ± 4.4	30.4 ± 3.5	28.9 ± 4.9	30.1 ± 3.7
Waist circumference, in cm, mean ± SD	95.4 ± 11.4	98.3 ± 11.1	95.8 ± 14.8	96.3 ± 13.5
Dutch MEDPED, in points, mean ± SD	9.1 ± 3.5 (n=16)	9.6 ± 3.7 (n=15)	8.7 ± 3 (n=13)	8.2 ± 4.3 (n=13)
HF diagnosis according to Dutch MEDPED, no./total no. (%)				
Probable (6-8 points)	10/16 (62.5%)	7/15 (46.7%)	8/13 (61.5%)	11/13 (84.6%)
Definitive (> 8 points)	6/16 (37.5%)	8/15 (53.3%)	5/13 (38.5%)	2/13 (15.4%)

[1]US$ = 5.70 Brazilian Reais

SD: standard deviation; FH: familial hypercholesterolemia; PP: phytosterol placebo; PK: krill oil placebo; P: active phytosterol; KO: active krill oil.

Table S5. Lipid-lowering therapy in use at baseline in the four study subgroups.

	**Control group (n=16)**	**PP/KO (n=15)**	**P/PK (n=13)**	**P/KO (n=14)**
Atorvastatin 20 mg	0/16 (0%)	1/15 (6.7%)	0/13 (0%)	1/14 (7.1%)
Atorvastatin 40 mg	0/16 (0%)	1/15 (6.7%)	0/13 (0%)	1/14 (7.1%)
Atorvastatin 40 mg + Ezetimibe 10 mg	1/16 (6.2%)	0/15 (0%)	0/13 (0%)	1/14 (7.1%)
Atorvastatin 60 mg	0/16 (0%)	1/15 (6.7%)	0/13 (0%)	0/14 (0%)
Atorvastatin 80 mg	3/16 (18.8%)	3/15 (20%)	2/13 (15.4%)	2/14 (14.3%)
Atorvastatin 80 mg + Ezetimibe 10 mg	7/16 (43.8%)	5/15 (33.3%)	6/13 (46.2%)	5/14 (35.7%)
Pitavastatin 4 mg	0/16 (0%)	1/15 (6.7%)	0/13 (0%)	0/14 (0%)
Rosuvastatin 10 mg	1/16 (6.2%)	0/15 (0%)	1/13 (7.7%)	1/14 (7.1%)
Rosuvastatin 20 mg	0/16 (0%)	0/15 (0%)	0/13 (0%)	1/14 (7.1%)
Rosuvastatin 20 mg + Ezetimibe 10 mg	0/16 (0%)	1/15 (6.7%)	1/13 (7.7%)	1/14 (7.1%)
Rosuvastatin 40 mg	1/16 (6.2%)	0/15 (0%)	0/13 (0%)	0/14 (0%)
Rosuvastatin 40 mg + Ezetimibe 10 mg	2/16 (12.5%)	0/15 (0%)	2/13 (15.4%)	1/14 (7.1%)
Simvastatin 40 mg	1/16 (6.2%)	2/15 (13.3%)	0/13 (0%)	0/14 (0%)
Other	0/16 (0%)	0/15 (0%)	1/13 (7.7%)	0/14 (0%)

PP: phytosterol placebo; P: phytosterol; KO: krill oil; PK: krill oil placebo.

Table S6. Genetic variants classified as pathogenic or of uncertain significance related to familial hypercholesterolemia identified in participants of the DICA-FH study.

Gene	Zygosity	Description (HGVS)	Location (GRCh38)
ABCG8	Homozygous	NM_022437.3: c.1608G>A (p.Trp536Ter)	2:g.43875265
APOB	Heterozygous	NM_000384.3: c.10580G>A (p.Arg3527Gln)	2:g.21006288
LDLR	Heterozygous	NM_000527.5: c.313+1G>A	19:g.11102787
LDLR	Heterozygous	NM_000527.5: c.501C>A (p.Cys167Ter)	19:g.11105407
LDLR	Heterozygous	NM_000527.5: c.530C>T (p.Ser177Leu)	19:g.11105436
LDLR	Heterozygous	NM_000527.5: c.818-2A>G	19:g.11107390
LDLR	Heterozygous	NM_000527.5: c.1060+10G>A	19:g.11110781
LDLR	Heterozygous	NM_000527.5: c.1118G>A (p.Gly373Asp)	19:g.11111571
LDLR	Heterozygous	NM_000527.5: c.1176C>A (p.Cys392Ter)	19:g.11111629
LDLR	Heterozygous	NM_000527.5: c.1216C>T (p.Arg406Trp)	19:g.11113307
LDLR	Heterozygous	NM_000527.5: c.1291G>A (p.Ala431Thr)	19:g.11113382
LDLR	Heterozygous	NM_000527.5: c.1352T>C (p.Ile451Thr)	19:g.11113443
LDLR	Heterozygous	NM_000527.5: c.1618G>A (p.Ala540Thr)	19:g.11116125
LDLR	Heterozygous	NM_000527.5: c.1775G>A (p.Gly592Glu)	19:g.11116928
LDLR	Heterozygous	NM_000527.5: c.1959del (p.Leu654SerfsTer11)	19:g.11120203
LDLR	Heterozygous	NM_000527.5: c.2041T>A (p.Cys681Ser)	19:g.11120423
LDLR	Heterozygous	NM_000527.5: c.2043C>A (p.Cys681Ter)	19:g.11120425
*LDLR**	Heterozygous	NM_000527.5: c.1587-3C>G	19:g.11116091
*LDLR**	Heterozygous	NM_000527.5: c.1801G>C (p.Asp601His)	19:g.11116954
*PCSK9**	Heterozygous	NM_174936.4: c.1486C>T (p.Arg496Trp)	1:g.55058630

HGVS: Human Genome Variation Society
*Variant of uncertain significance (VUS)
GRCh38: Genome Reference Consortium Human Build 38; *ABCG8*: ATP binding cassette subfamily G member 8 gene; *APOB*: Apolipoprotein B gene; *LDLR*: LDL receptor gene; *PCSK9*: Proprotein convertase subtilisin/kexin type 9 gene.

Methods and limitations

Genomic DNA obtained from a buccal swab sample was extracted and subjected to enrichment of coding regions (exome) through probe capture. These target regions were sequenced using the Illumina

NovaSeq 6000 genetic sequencer. FASTQ files were aligned to the GRCh38 (Genome Reference Consortium Human Build 38) reference genome using the DRAGEN Enrichment software. The VCF (Variant Call Format) file was annotated using the Emedgene software, and variants were filtered based on their population frequency and effect. The genes analyzed were selected according to their relevance to the investigated phenotype (familial hypercholesterolemia), following the guidelines of the ClinGen (Clinical Genome Resource). The identified variants were classified according to the criteria established by the American College of Medical Genetics and Genomics (ACMG). Only pathogenic, likely pathogenic, or variants of uncertain significance relevant to the participant's clinical findings were reported.

Complex structural variants, such as translocations, inversions, and repeat expansions, as well as copy number variations, were not analyzed. The method does not detect deep intronic variants, and the analysis was limited to intronic regions at splice sites. Exonic regions with low read coverage may contain variants not identified in this assay.

Table S7. Changes in plasma phytosterol biomarkers following the DICA-FH intervention, irrespective of study groups.

Phytosterol	Before (n= 20)	After (n= 20)	Difference 95%CI	P-value
Stigmasterol, μg/mL	0.16 [0.08 - 0.33]	0.09 [0.03 - 0.21]	-0.091 (-0.197; -0.001)	0.04
Stigmasterol, μg/mg TC*	0.08 [0.05 - 0.17]	0.05 [0.02 - 0.1]	-0.045 (-0.09; -0.001)	0.04
Beta-sitosterol, μg/mL	0.21 [0.12 - 1.7]	0.07 [0.02 - 0.12]	-0.81 (-1.17; -0.183)	<0.01
Beta-sitosterol, μg/mg TC*	0.1 [0.06 - 0.81]	0.03 [0.01 - 0.06]	-0.379 (-0.596; -0.073)	<0.01
Beta-sitosterol, μg/mg LDL-c*	0.16 [0.09 - 1.47]	0.05 [0.02 - 0.1]	-0.589 (-0.977; -0.117)	<0.01

TC: total cholesterol; LDL-c: low-density lipoprotein cholesterol

*Corrections for cholesterol were calculated using the ratio of sterol content (in μg) to cholesterol levels (either TC or LDL-c, in mg).

Table S8. Comparisons of erythrocyte fatty acids within and between study groups, before and after the intervention.

	**Phytosterol**			**Krill Oil**		
	**Placebo (n= 24)**	**Active (n= 23)**	**Difference 95%CI[1]**	**Placebo (n= 24)**	**Active (n= 23)**	**Difference 95%CI[1]**
**Caproic (C6:0), %**						
Baseline	1.02 [0.65 - 1.21]	0.73 [0.62 - 1.01]	-0.12 (-0.40; 0.13)	0.93 [0.63 - 1.18]	0.72 [0.64 - 1.19]	-0.03 (-0.31; 0.22)
Final	0.98 [0.72 - 1.64]	0.97 [0.72 - 1.53]	0.01 (-0.32; 0.38)	0.9 [0.66 - 1.64]	1.19 [0.82 - 1.41]	0.12 (-0.33; 0.44)
Difference (95%CI)[2]	0.13 (-0.18; 0.49)	0.29 (0.01; 0.67)	0.20 (-0.28; 0.59)	0.214 (-0.15; 0.55)	0.25 (-0.03; 0.59)	0.06 (-0.37; 0.50)
**Capric (C10:0), %**						
Baseline	0.06 [0.06 - 0.08]	0.07 [0.06 - 0.08]	0.00 (-0.01; 0.01)	0.07 [0.06 - 0.09]	0.06 [0.06 - 0.07]	0.00 (-0.02; 0.01)
Final	0.07 [0.05 - 0.08]	0.06 [0.05 - 0.06]	-0.01 (-0.02; 0.00)	0.06 [0.05 - 0.07]	0.06 [0.05 - 0.07]	0.00 (-0.01; 0.01)
Difference (95%CI)[2]	0 (-0.01; 0.01)	-0.01 (-0.02; 0.001)	-0.01 (-0.02; 0.01)	-0.01 (-0.03; 0.001)	-0.001 (-0.008; 0.006)	0.01 (-0.01; 0.02)
**Lauric (C12:0), %**						
Baseline	0.05 [0.04 - 0.08]	0.04 [0.04 - 0.05]	0.00 (-0.02; 0.01)	0.04 [0.04 - 0.07]	0.05 [0.04 - 0.06]	0.00 (-0.01; 0.01)
Final	0.03 [0.03 - 0.04]	0.04 [0.03 - 0.05]	0.01 (0.00; 0.02)	0.04 [0.03 - 0.04]	0.04 [0.03 - 0.05]	0.00 (-0.01; 0.01)
Difference (95%CI)[2]	-0.02 (-0.04; -0.01)*	-0.01 (-0.02; 0.002)	0.01 (0.00; 0.04)	-0.01 (-0.03; -0.005)*	-0.01 (-0.03; 0.00)‡	0.00 (-0.01; 0.02)
**Myristic (C14:0), %**						
Baseline	0.51 [0.35 - 0.8]	0.59 [0.39 - 0.77]	0.04 (-0.11; 0.22)	0.5 [0.36 - 0.72]	0.56 [0.4 - 0.8]	0.04 (-0.11; 0.21)
Final	0.48 [0.39 - 0.56]	0.45 [0.39 - 0.53]	0.00 (-0.09; 0.08)	0.44 [0.34 - 0.53]	0.49 [0.44 - 0.61]	0.09 (0.01; 0.16)¶
Difference (95%CI)[2]	-0.07 (-0.22; 0.03)	-0.11 (-0.30; 0.05)	-0.01 (-0.21; 0.15)	-0.11 (-0.27; 0.01)	-0.08 (-0.23; 0.06)	0.03 (-0.15; 0.22)
**Palmitic (C16:0), %**						
Baseline	22.8 [22.08 - 24.02]	22.56 [21.83 - 23.12]	-0.43 (-1.37; 0.37)	22.62 [21.97 - 23.33]	22.72 [21.7 - 23.6]	0.27 (-0.61; 1.16)
Final	21.08 [20.39 - 22.46]	21.29 [20.18 - 22.37]	-0.05 (-0.80; 0.70)	20.62 [20.06 - 22.3]	21.6 [20.64 - 22.77]	0.62 (-0.12; 1.44)
Difference (95%CI)[2]	-1.64 (-2.44; -0.95)*	-1.19 (-1.92; -0.61)*	0.46 (-0.58; 1.37)	-1.53 (-2.08; -0.89)*	-1.40 (-2.36; -0.55)*	0.15 (-0.86; 1.16)
**Palmitoleic (16:1), %**						

Baseline	0.42 [0.21 - 0.64]	0.51 [0.31 - 0.66]	0.07 (-0.11; 0.23)	0.48 [0.23 - 0.57]	0.46 [0.29 - 0.7]	0.07 (-0.12; 0.22)
Final	0.28 [0.22 - 0.51]	0.25 [0.21 - 0.43]	-0.04 (-0.16; 0.05)	0.24 [0.18 - 0.44]	0.26 [0.23 - 0.54]	0.05 (-0.02; 0.15)
Difference (95%CI)[2]	-0.11 (-0.24; 0.02)	-0.20 (-0.30; -0.07)*	-0.06 (-0.27; 0.09)	-0.17 (-0.28; -0.03)¶	-0.13 (-0.26; -0.02)≠	0.00 (-0.13; 0.20)
**Stearic (C18:0), %**						
Baseline	18.32 [17.47 - 19.71]	18.88 [17.24 - 19.65]	-0.10 (-1.05; 1.06)	18.32 [17.25 - 19.88]	18.88 [17.65 - 19.64]	0.06 (-0.96; 1.19)
Final	17.36 [16.42 - 18.64]	17.98 [17.15 - 19.03]	0.56 (-0.48; 1.54)	17.59 [16.61 - 18.81]	17.58 [16.22 - 18.52]	-0.36 (-1.33; 0.78)
Difference (95%CI)[2]	-0.99 (-1.99; -0.10)¶	-0.58 (-1.58; 0.22)	0.38 (-0.77; 1.67)	-0.48 (-1.30; 0.48)	-1.23 (-2.04; -0.32)*	-0.69 (-2.10; 0.53)
**Oleic (C18:1 n9c), %**						
Baseline	13.06 [12.31 - 14]	13.68 [12.98 - 15.47]	0.67 (-0.30; 1.84)	13.53 [12.28 - 15.61]	13.1 [12.87 - 13.89]	-0.18 (-1.31; 0.86)
Final	13.3 [12.35 - 15.26]	13.61 [12.21 - 14.43]	-0.06 (-1.48; 1.27)	13.5 [12.45 - 14.8]	13.46 [12.06 - 14.58]	-0.28 (-1.62; 1.09)
Difference (95%CI)[2]	-0.08 (-1.19; 1.71)	-0.63 (-1.40; 0.34)	-0.30 (-1.93; 0.93)	-0.45 (-1.39; 1.02)	-0.27 (-1.18; 1.30)	0.40 (-1.20; 1.63)
**Elaidic (C18:1 n9-trans), %**						
Baseline	1.12 [0.98 - 1.31]	1.21 [1.06 - 1.41]	0.07 (-0.07; 0.22)	1.18 [0.99 - 1.38]	1.18 [1.05 - 1.37]	0.03 (-0.14; 0.16)
Final	1.12 [1.07 - 1.19]	1.11 [1.02 - 1.2]	-0.01 (-0.10; 0.09)	1.1 [1.02 - 1.2]	1.12 [1.05 - 1.2]	0.02 (-0.08; 0.10)
Difference (95%CI)[2]	-0.08 (-0.16; 0.02)	-0.13 (-0.2; -0.08)*	-0.06 (-0.17; 0.02)	-0.11 (-0.17; -0.04)*	-0.10 (-0.2; -0.02)¶	0.01 (-0.09; 0.10)
**Linoleic (C18:2 n6c) + Linolelaidic (C18:2 n6-trans), %**						
Baseline	9.95 ± 2.15	10.68 ± 2.57	0.73 (-0.66; 2.13)	10.03 ± 2.5	10.6 ± 2.24	0.57 (-0.82; 1.96)
Final	9.79 ± 1.89	9.99 ± 2.43	0.19 (-1.09; 1.48)	9.53 ± 1.93	10.26 ± 2.34	0.73 (-0.53; 2.00)
Difference (95%CI)[2]	-0.16 (-0.93; 0.61)	-0.7 (-1.47; 0.07)	-0.54 (-1.60; 0.52)	-0.5 (-1.22; 0.22)	-0.34 (-1.18; 0.5)	0.16 (-0.91; 1.24)
**Arachidic (C20:0), %**						
Baseline	0.35 [0.1 - 0.41]	0.4 [0.3 - 0.44]	0.04 (-0.01; 0.12)	0.37 [0.24 - 0.42]	0.39 [0.19 - 0.43]	0.02 (-0.04; 0.08)
Final	0.05 [0.01 - 0.16]	0.08 [0.02 - 0.23]	0.02 (-0.02; 0.08)	0.07 [0.01 - 0.22]	0.06 [0.01 - 0.18]	0.00 (-0.05; 0.05)
Difference (95%CI)[2]	-0.17 (-0.26; -0.10)*	-0.19 (-0.30; -0.13)*	0.00 (-0.14; 0.07)	-0.17 (-0.28; -0.1)*	-0.19 (-0.30; -0.11)*	-0.02 (-0.11; 0.08)

**cis-11- Eicosenoic (C20:1), %**						
Baseline	0.21 [0.16 - 0.23]	0.22 [0.2 - 0.25]	0.02 (-0.02; 0.06)	0.22 [0.17 - 0.24]	0.22 [0.18 - 0.24]	0.00 (-0.03; 0.04)
Final	0.13 [0.05 - 0.18]	0.16 [0.06 - 0.23]	0.03 (-0.03; 0.10)	0.13 [0.08 - 0.21]	0.1 [0.02 - 0.2]	-0.03 (-0.10; 0.03)
Difference (95%CI)[2]	-0.09 (-0.17; -0.01)¶	-0.06 (-0.12; 0.01)	0.02 (-0.05; 0.13)	-0.05 (-0.12; 0.03)	-0.1 (-0.15; -0.02)*	-0.04 (-0.13; 0.03)
**cis-11,14- Eicosadienoic (C20:2), %**						
Baseline	0.14 [0.08 - 0.23]	0.23 [0.17 - 0.3]	0.08 (0.01; 0.14)	0.18 [0.09 - 0.27]	0.19 [0.09 - 0.25]	-0.01 (-0.07; 0.06)
Final	0.08 [0.04 - 0.1]	0.08 [0.06 - 0.15]	0.01 (-0.02; 0.04)	0.07 [0.04 - 0.1]	0.08 [0.06 - 0.15]	0.02 (-0.01; 0.05)
Difference (95%CI)[2]	-0.05 (-0.10; -0.01)≠	-0.10 (-0.17; -0.03)*	-0.04 (-0.13; 0.03)	-0.08 (-0.14; -0.03)*	-0.07 (-0.13; -0.003)‡	0.02 (-0.06; 0.10)
**cis-8,11,18 Eicosatrienoic (C20:3 n6), %**						
Baseline	1.61 ± 0.46	1.39 ± 0.33	-0.22 (-0.45; 0.02)	1.5 ± 0.47	1.51 ± 0.35	0.02 (-0.23; 0.26)
Final	1.32 ± 0.53	1.36 ± 0.41	0.04 (-0.24; 0.32)	1.29 ± 0.49	1.38 ± 0.47	0.09 (-0.19; 0.37)
Difference (95%CI)[2]	-0.29 (-0.49; -0.1)*	-0.04 (-0.17; 0.1)	0.25 (0.02; 0.48)¶	-0.2 (-0.4; -0.01)‡	-0.13 (-0.28; 0.02)	0.07 (-0.17; 0.31)
**Arachidonic (C20:4 n6), %**						
Baseline	16.48 [15.19 - 18.02]	16.77 [14.75 - 17.37]	-0.21 (-1.48; 1.21)	16.54 [14.86 - 17.63]	16.66 [15.29 - 17.78]	0.24 (-1.21; 1.50)
Final	16.18 [14.19 - 16.84]	15.93 [15.11 - 16.95]	-0.12 (-1.16; 1.06)	16.18 [14.98 - 16.87]	15.77 [14.16 - 16.95]	-0.34 (-1.46; 0.71)
Difference (95%CI)[2]	-0.66 (-1.3; 0.14)	-0.57 (-1.24; 0.20)	0.12 (-0.90; 1.14)	-0.40 (-1.01; 0.30)	-0.84 (-1.57; 0.03)	-0.54 (-1.39; 0.55)
**cis-11,14,17 Eicosatrienoic (C20:3 n3), %**						
Baseline	0.04 [0.02 - 0.18]	0.06 [0.03 - 0.09]	0.01 (-0.02; 0.03)	0.05 [0.03 - 0.1]	0.04 [0.03 - 0.14]	0.00 (-0.03; 0.02)
Final	0.03 [0.02 - 0.14]	0.04 [0.02 - 0.16]	0.01 (-0.02; 0.04)	0.03 [0.02 - 0.12]	0.04 [0.02 - 0.17]	0.01 (-0.02; 0.04)
Difference (95%CI)[2]	-0.01 (-0.16; 0.09)	0.01 (-0.03; 0.18)	0.02 (-0.05; 0.21)	-0.01 (-0.40; 0.10)	0 (-0.05; 0.13)	0.01 (-0.08; 0.12)
**Behenic (C22:0), %**						
Baseline	1.37 [1.06 - 1.47]	1.44 [1.29 - 1.56]	0.04 (-0.10; 0.24)	1.37 [1.26 - 1.51]	1.46 [1.18 - 1.5]	0.02 (-0.17; 0.17)
Final	1.23 [0.06 - 1.79]	0.66 [0.08 - 1.56]	-0.03 (-1.01; 0.18)	1.41 [0.08 - 1.9]	0.23 [0.05 - 1.42]	-0.13 (-1.09; 0.10)
Difference (95%CI)[2]	-0.18 (-0.52; 0.30)	-0.32 (-0.81; 0.31)	-0.21 (-0.87; 0.47)	-0.01 (-0.54; 0.53)	-0.41 (-0.81; 0.07)	-0.32 (-0.91; 0.25)

**Erucic (C22:1 n9), %**						
Baseline	0.17 [0.03 - 0.29]	0.16 [0.1 - 0.25]	0.00 (-0.09; 0.10)	0.17 [0.05 - 0.26]	0.16 [0.1 - 0.21]	-0.01 (-0.10; 0.10)
Final	2.85 [2.06 - 4.77]	2.9 [1.21 - 4.53]	-0.21 (-2.08; 0.92)	3.07 [2.04 - 4.52]	2.81 [1.94 - 4.65]	-0.13 (-1.83; 1.10)
Difference (95%CI)[2]	2.78 (1.39; 4.38)*	2.51 (1.31; 3.74)*	-0.10 (-2.29; 1.56)	2.83 (1.42; 4.24)*	2.56 (1.29; 3.84)*	-0.11 (-2.06; 1.64)
**cis-13,16 Docosadienoic (C22:2), %**						
Baseline	0.08 [0.05 - 0.13]	0.11 [0.06 - 0.18]	0.03 (-0.01; 0.08)	0.1 [0.05 - 0.16]	0.09 [0.06 - 0.15]	0.00 (-0.04; 0.04)
Final	0.08 [0.04 - 0.12]	0.1 [0.08 - 0.13]	0.01 (-0.02; 0.05)	0.1 [0.08 - 0.14]	0.08 [0.03 - 0.11]	-0.03 (-0.07; 0.00)
Difference (95%CI)[2]	-0.01 (-0.05; 0.03)	-0.03 (-0.16; 0.02)	-0.01 (-0.08; 0.05)	0 (-0.06; 0.04)	-0.04 (-0.11; 0.01)	-0.03 (-0.09; 0.02)
**Lignoceric (C24:0), %**						
Baseline	3.73 [3.07 - 3.93]	3.56 [3.23 - 3.89]	-0.11 (-0.49; 0.26)	3.71 [3.09 - 4.37]	3.63 [3.27 - 3.86]	-0.14 (-0.65; 0.19)
Final	3.57 [3.31 - 4.19]	3.45 [3.1 - 3.95]	-0.16 (-0.55; 0.20)	3.64 [3.41 - 4.36]	3.36 [3.13 - 3.8]	-0.29 (-0.66; 0.06)
Difference (95%CI)[2]	0.04 (-0.38; 0.52)	-0.08 (-0.48; 0.32)	-0.12 (-0.69; 0.51)	0.02 (-0.45; 0.59)	-0.04 (-0.43; 0.29)	-0.11 (-0.70; 0.57)
**Nervonic (C24:1), %**						
Baseline	2.86 [2.63 - 3.03]	2.82 [2.58 - 3.26]	0.02 (-0.27; 0.32)	2.86 [2.67 - 3]	2.73 [2.31 - 3.29]	-0.08 (-0.37; 0.26)
Final	3.05 [2.37 - 3.86]	3.2 [2.53 - 3.7]	0.12 (-0.51; 0.63)	3.14 [2.75 - 3.69]	2.76 [2.3 - 3.9]	-0.19 (-0.80; 0.49)
Difference (95%CI)[2]	0.28 (-0.02; 0.73)	0.35 (0.03; 0.88)‡	0.10 (-0.42; 0.50)	0.31 (-0.003; 0.71)	0.31 (0.02; 0.91)•	-0.01 (-0.46; 0.52)

[1] Difference in medians between groups (active – placebo) using the Hodges-Lehmann estimator; for linoleic (C18:2 n6c) + linolelaidic (C18:2 n6-trans), and cis-8,11,18 eicosatrienoic (C20:3 n6) fatty acids differences in mean percentage between groups (active – placebo) using the non-paired Student´s T test.

[2] Intra-group median difference by the paired Wilcoxon test; 95% confidence interval (95% CI) estimated for the median of the differences between paired observations; for linoleic (C18:2 n6c) + linolelaidic (C18:2 n6-trans), and cis-8,11,18 eicosatrienoic (C20:3 n6), intra-group mean difference by the paired t test; 95% CI estimated for the mean of the differences between paired observations.

*P= <0.01; ¶P= 0.03; ≠P= 0.02; •P= 0.01; ‡P= 0.04

Table S9. Changes in erythrocyte fatty acids following the DICA-FH intervention, irrespective of study groups.

Fatty acid, in %	Before (n= 47)	After (n= 47)	Difference 95%CI	P-value
Caproic (C6:0)	0.82 [0.63 - 1.19]	0.97 [0.7 - 1.61]	0.225 (0.008; 0.457)	0.04
Lauric (C12:0)	0.05 [0.04 - 0.07]	0.04 [0.03 - 0.05]	-0.013 (-0.023; -0.006)	< 0.01
Palmitic (C16:0)	22.63 [21.83 - 23.4]	21.18 [20.3 - 22.44]	-1.433 (-1.954; -0.967)	< 0.01
Palmitoleic (C16:1)	0.48 [0.26 - 0.66]	0.25 [0.21 - 0.48]	-0.158 (-0.234; -0.057)	< 0.01
Stearic (C18:0)	18.32 [17.34 - 19.68]	17.58 [16.54 - 18.74]	-0.767 (-1.496; -0.199)	< 0.01
Elaidic (C18:1 n9-trans)	1.18 [1.04 - 1.39]	1.11 [1.02 - 1.2]	-0.104 (-0.15; -0.058)	< 0.01
cis-11-Eicosenoic (C20:1)	0.22 [0.18 - 0.24]	0.13 [0.05 - 0.2]	-0.071 (-0.116; -0.017)	< 0.01
cis-11,14-Eicosadienoic (C20:2)	0.19 [0.09 - 0.26]	0.08 [0.05 - 0.11]	-0.074 (-0.116; -0.032)	< 0.01
cis-8,11,14-Eicosatrienoic (C20:3 n6)	1.51 ± 0.41	1.34 ± 0.47	-0.17 (-0.29; -0.048)	< 0.01
Arachidonic (C20:4 n6)	16.66 [15.15 - 17.77]	15.99 [14.33 - 16.95]	-0.613 (-1.095; -0.09)	0.03
Erucic (C22:1 n9)	0.16 [0.05 - 0.25]	2.9 [1.94 - 4.55]	2.658 (1.668; 3.59)	< 0.01
Docosahexaenoic (DHA, C22:6 n3)	3.45 ± 0.96	4.39 ± 1.31	0.95 (0.55; 1.343)	< 0.01
Nervonic (C24:1)	2.85 [2.6 - 3.13]	3.07 [2.4 - 3.73]	0.324 (0.107; 0.608)	< 0.01

Table S10. Comparisons of clinical lipid profile markers within and between study groups, before and after the intervention.

	Phytosterol			Krill Oil		
	**Placebo (n= 26)**	**Active (n= 24)**	**Difference 95%CI[1]**	**Placebo (n= 25)**	**Active (n= 25)**	**Difference 95%CI[1]**
**TC, mg/dL**						
Baseline	208.5 [183.8 - 221]	196 [164.8 - 231.8]	-9 (-33; 18)	190 [152 - 212]	211 [194 - 240]	28 (4; 59)
Final	186.5 [164 - 224]	192 [161.8 - 225.3]	-0.6 (-33; 33)	191 [158 - 226]	189 [171 - 225]	17 (-15; 53)
Difference, in mg/dL (95%CI)[2]	-12 (-26.5; 6)	-3.917 (-24; 19.5)	3.6 (-18; 29.9)	-1.8 (-18; 12)	-14 (-32; 17.5)	-12 (-32; 9.2)
Difference, in % (IQR)[3]	-4.1 [-11.5 - 0]	-5.6 [-17.3 - 16]	1 (-9.3; 15.6)	-0.6 [-10.5 - 13.9]	-7.1 [-18.2 - 0.4]	-5.2 (-15.6; 7.1)
**HDL-c, mg/dL**						
Baseline	49 ± 12	54.2 ± 11.8	5.2 (-1.6; 11.9)	50.7 ± 12.9	52.3 ± 11.4	1.6 (-5.3; 8.5)
Final	47.3 ± 11.6	48.7 ± 10.5	1.4 (-4.9; 7.7)	45.7 ± 11.3	50.3 ± 10.4	4.5 (-1.6; 10.7)
Difference, in mg/dL (95%CI)[2]	-1.7 (-5.5; 2.1)	-5.5 (-8.3; -2.7)*	-3.8 (-8.4; 0.9)	-5 (-9.3; -0.7)≠	-2.1 (-4.3; 0.2)	2.9 (-1.8; 7.7)
Difference, in % (SD)[3]	-2.2 (16.3)	-9.3 (11.3)	-7.1 (-15.1; 0.8)	-7.8 (18.1)	-3.4 (9.4)	4.4 (-3.9; 12.7)
**Triglycerides, mg/dL**						
Baseline	113.5 [93.5 - 142]	97 [80.5 - 165]	-11.6 (-36; 21)	102 [82 - 124]	118.5 [87.85 - 171.8]	12 (-15; 44)
Final	102 [85.5 - 160]	118 [77.5 - 174]	-2 (-34; 32)	118 [79 - 157]	107 [82.5 – 164]	0.00 (-32; 33)
Difference, in mg/dL (95%CI)[2]	-3.5 (-25.6; 13)	-9.1 (-32.5; 16)	-6 (-26; 25)	0 (-23.5; 28.5)	-8.7 (-40.5; 6.5)	-10 (-42.9; 14)
Difference, in % (IQR)[3]	3.9 [-27.4 - 18.2]	-8.2 [-19.8 - 18.5]	-3.6 (-22; 19.8)	0 [-20.8 - 30.2]	-5.2 [-24.1 - 9.4]	-8.2 (-30; 11.2)
**VLDL-c, mg/dL**						
Baseline	22.7 [18.7 - 28.4]	19.4 [16.1 - 33]	-2.3 (-7.2; 4.2)	20.4 [16.4 - 24.8]	23.7 [17.6 - 34.4]	2.4 (-3; 8.8)
Final	20.4 [17.1 - 32]	23.6 [15.5 - 34.8]	-0.4 (-6.8; 6.4)	23.6 [15.8 - 31.4]	21.4 [16.5 - 32.8]	0.00 (-6.4; 6.6)
Difference, in mg/dL (95%CI)[2]	-0.7 (-5.1; 2.6)	-1.8 (-6.5; 3.2)	-1.2 (-5.2; 5)	0 (-4.7; 5.7)	-1.7 (-8.1; 1.3)	-2 (-8.6; 2.8)
Difference, in % (IQR)[3]	3.9 [-27.4 - 18.2]	-8.2 [-19.8 - 18.5]	-3.6 (-22; 19.8)	0 [-20.8 - 30.2]	-5.2 [-24.1 - 9.4]	-8.2 (-30; 11.2)
**Non-HDL-c, mg/dL**						
Baseline	154 [129 - 176.4]	138.1 [121.5 - 171.5]	-11.7 (-38; 20)	135 [100 - 173]	155 [136 - 195]	28 (3; 58)
Final	144.5 [112.5 - 167.8]	148.8 [114 - 184.8]	1 (-38; 32.5)	137 [98 - 182]	152 [120.9 - 182]	15 (-18; 52)
Difference, in mg/dL (95%CI)[2]	-9.9 (-23; 7)	1.7 (-16.5; 25.5)	8 (-12; 30.5)	2 (-11; 16)	-12.6 (-27.5; 19.5)	-16 (-33; 5)

Difference, in % (IQR)[3]	-5.4 [-18.9 - 3.4]	-0.5 [-18.1 - 25.5]	4 (-10.6; 22.1)	0 [-11.7 - 17.8]	-8.9 [-19.7 - 0.9]	-7.8 (-21.8; 7.4)
**TC/HDL-c ratio, mg/dL**						
Baseline	3.8 [3.6 - 5.4]	3.5 [3 - 4.5]	-0.4 (-1.2; 0.2)	3.7 [3.3 - 4.7]	4.2 [3.3 - 5.5]	0.5 (-0.3; 1.2)
Final	4.2 [3.2 - 5.3]	3.9 [3.2 - 5.4]	-0.2 (-1.1; 0.7)	4.2 [3.3 - 5.3]	4 [3.2 - 5.6]	0.1 (-0.8; 1.1)
Difference, in mg/dL (95%CI)[2]	-0.1 (-0.5; 0.5)	0.4 (0.03; 0.7)¶	0.5 (-0.01; 1)	0.44 (-0.02; 1)	-0.1 (-0.4; 0.4)	-0.4 (-0.9; 0.1)
Difference, in % (IQR)[3]	-4.5 [-15.1 - 12.5]	6.7 [-5.2 - 29.6]	11.83 (-1.35; 24.11)	9.6 [-8.2 - 27.8]	-4.9 [-11.1 - 5]	-10.3 (-23.8; 3.6)
**LDL-c/HDL-c ratio, mg/dL**						
Baseline	2.5 [2.1 - 3.7]	2 [1.7 - 3]	-0.4 (-1; 0.1)	2.2 [1.7 - 3]	2.8 [1.9 - 3.9]	0.5 (-0.2; 1.2)
Final	2.7 [1.9 - 3.7]	2.6 [1.7 - 3.9]	-0.2 (-1.1; 0.7)	2.6 [1.6 - 3.6]	2.8 [1.8 - 3.9]	0.2 (-0.7; 1.1)
Difference, in mg/dL (95%CI)[2]	-0.1 (-0.4; 0.4)	0.4 (-0.02; 0.8)	0.4 (-0.1; 0.8)	0.3 (-0.08; 0.9)	-0.1 (-0.4; 0.4)	-0.4 (-0.8; 0.1)
Difference, in % (IQR)[3]	-3.6 [-20.8 - 13.9]	4.9 [-7.5 - 41.2]	13.6 (-7.4; 35.6)	13.1 [-4.1 - 37.5]	-5.3 [-16.6 - 6.4]	-13.1 (-31.5; 6.8)
**TG/HDL-c ratio, mg/dL**						
Baseline	2.4 [1.52 - 3.5]	1.9 [1.4 - 2.5]	-0.3 (-1.1; 0.2)	2 [1.4 - 2.7]	2.3 [1.5 - 3.6]	0.1 (-0.5; 0.9)
Final	2.5 [1.5 - 3.5]	2.3 [1.4 - 4.2]	-0.2 (-1; 0.8)	2.4 [1.6 - 4.1]	2.3 [1.5 - 3.6]	-0.1 (-1.1; 0.6)
Difference, in mg/dL (95%CI)[2]	0.1 (-0.5; 0.4)	0.2 (-0.3; 0.7)	0.1 (-0.4; 0.8)	0.4 (-0.1; 1)	-0.1 (-0.8; 0.2)	-0.5 (-1.1; 0.03)
Difference, in % (IQR)[3]	6.3 [-20.3 – 24]	0.7 [-7 - 36.1]	5.5 (-15.2; 31.3)	12.1 [-4.7 - 48.9]	-0.3 [-17.4 - 20]	-17.4 (-41.8; 2)
**Atherogenic Index, mg/dL**						
Baseline	2.8 [2.6 - 4.4]	2.5 [2 - 3.5]	-0.4 (-1.2; 0.2)	2.7 [2.3 - 3.7]	3.2 [2.3 - 4.5]	0.5 (-0.3; 1.2)
Final	3.2 [2.2 - 4.3]	2.9 [2.2 - 4.4]	-0.2 (-1.1; 0.7)	3.2 [2.3 - 4.3]	3 [2.2 - 4.6]	0.1 (-0.8; 1.1)
Difference, in mg/dL (95%CI)[2]	-0.1 (-0.5; 0.5)	0.4 (0.03; 0.9)¶	0.5 (-0.01; 1)	0.4 (-0.02; 1)	-0.1 (-0.4; 0.4)	-0.4 (-0.9; 0.1)
Difference, in % (IQR)[3]	-5.5 [-20.2 - 17.1]	8.8 [-6.6 - 39.6]	15.8 (-3; 32.5)	13.5 [-10.1 - 39.4]	-5.8 [-15.1 - 7.7]	-13.3 (-31.7; 6.8)

[1] Difference in medians between groups (active – placebo) using the Hodges-Lehmann estimator; for high density lipoprotein cholesterol (HDL-c), difference in means between groups (active – placebo) using the non-paired Student´s T test.

[2] Intra-group median difference by the paired Wilcoxon test; 95% confidence interval (95% CI) estimated for the median of the differences between paired observations; for HDL-c, intra-group mean difference by the paired t test; 95% CI estimated for the mean of the differences between paired observations.

[3] Percentual difference in intra-group medians or means, calculated as: 100 * (final– initial / final) .

TC: total cholesterol; VLCL-c: very low-density lipoprotein cholesterol; TG: triglycerides; LDL-c: low-density lipoprotein cholesterol; SD: standard deviation; IQR: interquartile range.

*P< 0.01; ¶P= 0.04; ≠P= 0.02

Table S11. Comparisons of oxidized low density lipoprotein cholesterol and apolipoproteins within and between study groups, before and after the intervention.

	Phytosterol			Krill Oil		
	**Placebo (n= 26)**	**Active (n= 24)**	**Difference 95%CI[1]**	**Placebo (n= 25)**	**Active (n= 25)**	**Difference 95%CI[1]**
**oxLDL, mg/dL**						
Baseline	0.18 [0.16 - 0.23]	0.21 [0.13 - 0.26]	0.01 (-0.05; 0.06)	0.2 [0.14 - 0.22]	0.2 [0.16 - 0.3]	0.03 (-0.03; 0.09)
Final	0.15 [0.11 - 0.2]	0.18 [0.1 - 0.25]	0.02 (-0.03; 0.07)	0.15 [0.1 - 0.2]	0.16 [0.12 - 0.25]	0.02 (-0.03; 0.06)
Difference, in mg/dL (95%CI)[2]	-0.04 (-0.08; -0.01)*	-0.03 (-0.07; -0.001)¶	0.01 (-0.03; 0.05)	-0.03 (-0.06; 0.003)	-0.04 (-0.08; -0.01)•	-0.01 (-0.06; 0.03)
Difference, in % (IQR)[3]	-19.8 [-36 - 2.3]	-13.8 [-31.6 - 2.8]	5.1 (-14.1; 24.1)	-17 [-33.9 - 11.1]	-15.1 [-35.3 - 0.71]	-0.21 (-21.5; 18.5)
**oxLDL/LDL-c ratio, mg/dL**						
Baseline	0.16 [0.13 - 0.2]	0.16 [0.13 - 0.2]	0.01 (-0.03; 0.04)	0.16 [0.14 - 0.2]	0.16 [0.11 - 0.2]	-0.01 (-0.05; 0.03)
Final	0.12 [0.1 - 0.2]	0.15 [0.12 - 0.16]	0.02 (-0.02; 0.04)	0.13 [0.11 - 0.16]	0.13 [0.10 - 0.17]	-0.01 (-0.04; 0.02)
Difference, in mg/dL (95%CI)[2]	-0.03 (-0.05; -0.003)≠	-0.03 (-0.07; 0.001)	-0.003 (-0.04; 0.04)	-0.03 (-0.06; 0.002)	-0.03 (-0.06; -0.005)*	0.001 (-0.04; 0.04)
Difference, in % (SD)[3]	-11.7 (35.1)	-9.5 (35.3)	2.2 (-18.1; 22.5)	-9.7 (39.4)	-11.6 (30.2)	-2 (-22.1; 18.2)
**Apo B, mg/dL**						
Baseline	114.5 [69 - 208.8]	157.5 [84.5 - 282.5]	18 (-32; 92)	126 [66 - 263]	127 [86 - 210]	1 (-74; 46)
Final	131.5 [77 - 237.5]	121 [91.5 - 213.5]	5 (-53; 49)	138 [85 - 236]	114 [77 - 238]	-9 (-69; 41)
Difference, in mg/dL (95%CI)[2]	9.5 (-5.5; 28.5)	-3.2 (-38; 22)	-13 (-41; 13)	2.5 (-15.5; 29)	7 (-14.5; 27)	6 (-23; 30)
Difference, in % (IQR)[3]	7.8 [-8.9 - 19]	-0.2 [-14.5 - 15]	-6.8 (-26.8; 10.1)	2.7 [-14.3 - 12.5]	8.6 [-14.2 - 19.9]	2.1 (-20.1; 20.2)
**Apo A-I, mg/dL**						
Baseline	115 [67.8 - 140.3]	144.5 [108 - 162.5]	30 (1; 65)	126 [76 - 145]	127 [84 - 158]	8 (-26; 39)
Final	91 [68.8 - 163.8]	123.5 [88 - 156.8]	21.4 (-8; 52)	98 [71 - 131]	103 [76 - 184]	11 (-19; 57)
Difference, in mg/dL (95%CI)[2]	1 (-21; 27)	-8 (-30; 10.5)	-14 (-41; 19)	-8.5 (-37.5; 14)	1.5 (-17.5; 21.5)	11 (-17; 43)
Difference, in % (IQR)[3]	0.3 [-17.5 - 30.9]	-13.2 [-26.9 - 26.3]	-11.2 (-32.5; 16.2)	-12 [-31.9 - 31.5]	-1 [-19 - 25]	3.1 (-20.1; 27.9)
**Apo B/Apo A-I ratio, mg/dL**						
Baseline	1.6 [0.8 - 2.4]	1.3 [0.6 - 2]	-0.2 (-0.9; 0.3)	1.7 [0.7 - 2.5]	1.1 [0.61 - 1.8]	-0.2 (-1; 0.3)
Final	1.5 [0.8 - 2.7]	1.3 [0.7 - 2.1]	-0.3 (-0.9; 0.4)	1.5 [0.8 - 2.8]	1.2 [0.7 - 1.9]	-0.3 (-1; 0.4)
Difference, in mg/dL (95%CI)[2]	-0.15 (-0.32; 0.42)	0.03 (-0.24; 0.3)	0.13 (-0.32; 0.46)	-0.06 (-0.35; 0.39)	-0.02 (-0.24; 0.26)	0.1 (-0.3; 0.4)

Difference, in % (IQR)[3]	-8.8 [-25.3 - 56.8]	6.5 [-21.6 - 49.2]	6.9 (-28.9; 33.2)	-5.6 [-27.8 - 50.9]	-4.7 [-21.4 - 48.7]	3.5 (-27.5; 29.2)

[1] Difference in medians between groups (active – placebo) using the Hodges-Lehmann estimator; for oxidized low density lipoprotein cholesterol/low density lipoprotein cholesterol (oxLDL/LDL-c) ratio, difference in mean percentage between groups (active – placebo) using the non-paired Student´s T test.

[2] Intra-group median difference by the paired Wilcoxon test; 95% confidence interval (95% CI) estimated for the median of the differences between paired observations.

[3] Percentual difference in intra-group medians or means, calculated as: 100 * (final– initial / final).

Apo: apolipoprotein; SD: standard deviation; IQR: interquartile range.

*P= 0.02; ¶P= 0.04; ≠P= 0.03; •P= 0.01

Table S12. Adverse events reported during the study in the four study subgroups.

Adverse event	Control group (n=16)	PP/KO (n=15)	P/PK (n=13)	P/KO (n=14)
Nasopharyngitis	6/16 (37.5%)	5/15 (33.3%)	4/13 (30.8%)	5/14 (35.7%)
Pyrosis	6/16 (37.5%)	5/15 (33.3%)	2/13 (15.4%)	3/14 (21.4%)
Eructation	6/16 (37.5%)	3/15 (20%)	2/13 (15.4%)	3/14 (21.4%)
Abdominal discomfort	5/16 (31.2%)	6/15 (40%)	2/13 (15.4%)	2/14 (14.3%)
Headache	5/16 (31.2%)	3/15 (20%)	3/13 (23.1%)	1/14 (7.1%)
Residual taste perception	3/16 (18.8%)	6/15 (40%)	2/13 (15.4%)	2/14 (14.3%)
Nausea	5/16 (31.2%)	2/15 (13.3%)	2/13 (15.4%)	2/14 (14.3%)
Diarrhea	4/16 (25%)	3/15 (20%)	0/13 (0%)	2/14 (14.3%)
Loss of appetite	2/16 (12.5%)	1/15 (6.7%)	4/13 (30.8%)	3/14 (21.4%)
Epigastric pain	3/16 (18.8%)	4/15 (26.7%)	1/13 (7.7%)	0/14 (0%)
Halitosis	2/16 (12.5%)	2/15 (13.3%)	0/13 (0%)	2/14 (14.3%)
Cutaneous manifestations of hypersensitivity	1/16 (6.2%)	3/15 (20%)	1/13 (7.7%)	1/14 (7.1%)
Vomiting	1/16 (6.2%)	1/15 (6.7%)	1/13 (7.7%)	1/14 (7.1%)
Coronavirus 2019 infection	1/16 (6.2%)	0/15 (0%)	2/13 (15.4%)	0/14 (0%)
Alterations in serum enzyme levels	0/16 (0%)	0/15 (0%)	1/13 (7.7%)	0/14 (0%)
Cardiac arrhythmia	0/16 (0%)	1/15 (6.7%)	0/13 (0%)	0/14 (0%)
Colicky pain	0/16 (0%)	1/15 (6.7%)	0/13 (0%)	0/14 (0%)
Constipation	0/16 (0%)	0/15 (0%)	2/13 (15.4%)	1/14 (7.1)
Myalgia and/or arthralgia	1/16 (6.2%)	2/15 (13.3%)	1/13 (7.7%)	1/14 (7.1%)
Chest pain	1/16 (6.2%)	0/15 (0%)	0/13 (0%)	0/14 (0%)
Heart failure	0/16 (0%)	0/15 (0%)	0/13 (0%)	1/14 (7.1%)
Sudden cardiac death	0/16 (0%)	0/15 (0%)	0/13 (0%)	1/14 (7.1%)
Gastrointestinal hemorrhage	0/16 (0%)	0/15 (0%)	1/13 (7.7%)	0/14 (0%)
Anxiety disorder	1/16 (6.2%)	0/15 (0%)	0/13 (0%)	0/14 (0%)
Blurred vision	1/16 (6.2%)	0/15 (0%)	0/13 (0%)	0/14 (0%)

PP: phytosterol placebo; PK: krill oil placebo; P: active phytosterol; KO: active krill oil.

Table S13. Comparisons of liver enzymes within and between study groups, before and after the intervention.

	Phytosterol			Krill Oil		
	**Placebo (n= 26)**	**Active (n= 24)**	**Difference 95%CI[1]**	**Placebo (n= 25)**	**Active (n= 25)**	**Difference 95%CI[1]**
**Aspartate aminotransferase, U/L**						
Baseline	26 [23 - 34.75]	23.5 [21 - 28.25]	-3.00 (-8.00; 1.00)	26 [22 - 35]	24 [21 - 27]	-3.00 (-7.00; 2.00)
Final	27 [21.25 - 30]	23.5 [20 - 31]	-1.00 (-6.00; 3.00)	23 [20 - 33]	27 [21 - 30]	1.00 (-4.00; 5.00)
Difference, in U/L (95%CI)[2]	-1.8 (-5; 1.5)	0.5 (-3.5; 6)	2.00 (-3.00; 7.00)	-2.5 (-6.5; 2)	1 (-2.5; 4.5)	3.00 (-2.00; 8.00)
**Alanine aminotransferase, U/L**						
Baseline	31.5 [24.5 - 50.25]	27.5 [22 - 32]	-6.00 (-16.00; 1.00)	32 [22 - 48]	27 [22 - 32]	-5.00 (-16.00; 2.00)
Final	28.5 [22.75 - 42]	26 [19 - 36.25]	-3.00 (-10.00; 4.00)	26 [24 - 44]	28 [20 - 35]	-2.00 (-9.00; 5.00)
Difference, in U/L (95%CI)[2]	-1.5 (-9.5; 2.5)	1.5 (-4.5; 8.5)	3.00 (-3.00; 11.00)	-4.468 (-14; 2.5)	2 (-1.5; 5.5)	5.00 (-2.00; 14.00)

[1] Difference in medians between groups (active – placebo) using the Hodges-Lehmann estimator.

[2] Intra-group median difference by the paired Wilcoxon test; 95% confidence interval (95% CI) estimated for the median of the differences between paired observations.
